# Supplementary material for: ERK1/2 inhibition promotes robust myotube growth via CaMKII activation resulting in myoblast-to-myotube fusion
Source: Dev Cell. 2021 Dec 20;56(24):3349–3363.e6. doi: 10.1016/j.devcel.2021.11.022 (PMC8693863; doi:10.1016/j.devcel.2021.11.022)
Supplement: Document S1. Figures S1–S7 and Tables S1 and S2 [file mmc1.pdf]

Developmental Cell, Volume *56*

Supplemental information

ERK1/2 inhibition promotes robust myotube growth via CaMKII activation resulting in myoblast-to-myotube fusion

Tamar Eigler, Giulia Zarfati, Emmanuel Amzallag, Sansrity Sinha, Nadav Segev, Yishaia Zabary, Assaf Zaritsky, Avraham Shakked, Kfir-Baruch Umansky, Eyal D. Schejter, Douglas P. Millay, Eldad Tzahor, and Ori Avinoam

Supplemental Information

ERK1/2 inhibition promotes robust myotube growth via CaMKII activation resulting in myoblast-to-myotube fusion

Tamar Eigler[1], Giulia Zarfati[2], Emmanuel Amzallag[1], Sansrity Sinha[2], Nadav Segev[2], Yishaia Zabary[3], Assaf Zaritsky[3], Avraham Shakked[1], Kfir-Baruch Umansky[1], Eyal D. Schejter[4], Douglas P. Millay[5,6], Eldad Tzahor[1*†] and Ori Avinoam[2*]

Affiliations:

[1]Department of Molecular Cell Biology, Weizmann Institute of Science, Rehovot, Israel.

[2]Department of Biomolecular Sciences, Weizmann Institute of Science, Rehovot, Israel.

[3]Department of Software & Information Systems Engineering, Ben Gurion University, Be’er Sheva, Israel.

[4]Department of Molecular Genetics, Weizmann Institute of Science, Rehovot, Israel.

[5]Division of Molecular Cardiovascular Biology, Cincinnati Children's Hospital Medical Center, Cincinnati, Ohio, USA.

[6]Department of Pediatrics, University of Cincinnati College of Medicine, Cincinnati, Ohio, USA.

* Both authors share corresponding authorship: eldad.tzahor@weizmann.ac.il and ori.avinoam@weizmann.ac.il

† Lead contact

Supplemental Figure 1: ERKi inhibition in DM enhances differentiation and fusion, related to figure 1

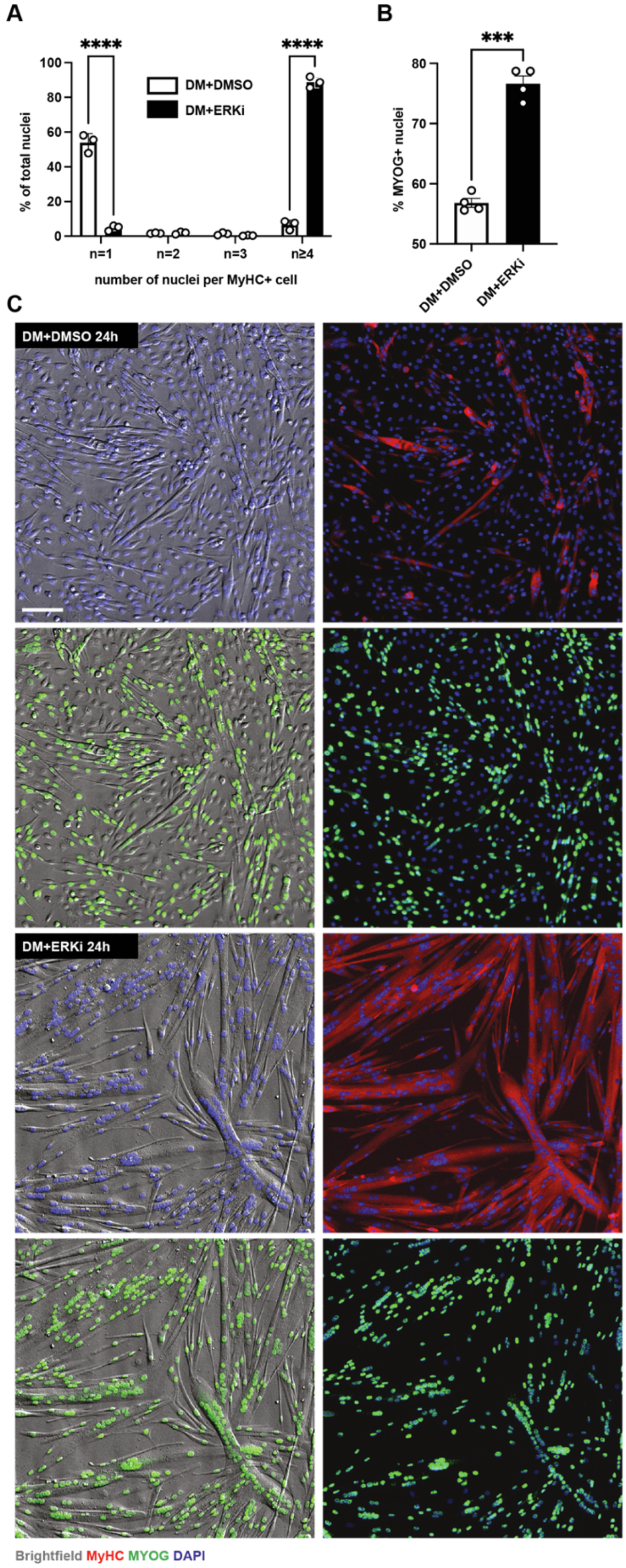

Supplemental Figure 1:

(**A)** Stratified fusion index of myoblasts co-treated with DM and ERKi , compared to DM treatment alone for 48 hours. (**B**) Quantification of MYOG positive nuclei per field as shown in **C**, using a semi-automated image analysis script to segment and overlap MyoG positive nuclei with total nuclei in an unbiased manner. See materials and methods for details. (**C**) Representative images of the IF staining for MYOG (green) and their correlation with nuclei (blue), and MyHC (red). Scale bars, 100 μm.

Supplemental Figure 2: ERKi induced fusion is not recapitulated by simulation studies of random fusion or weighted fusion, related to Figure 2

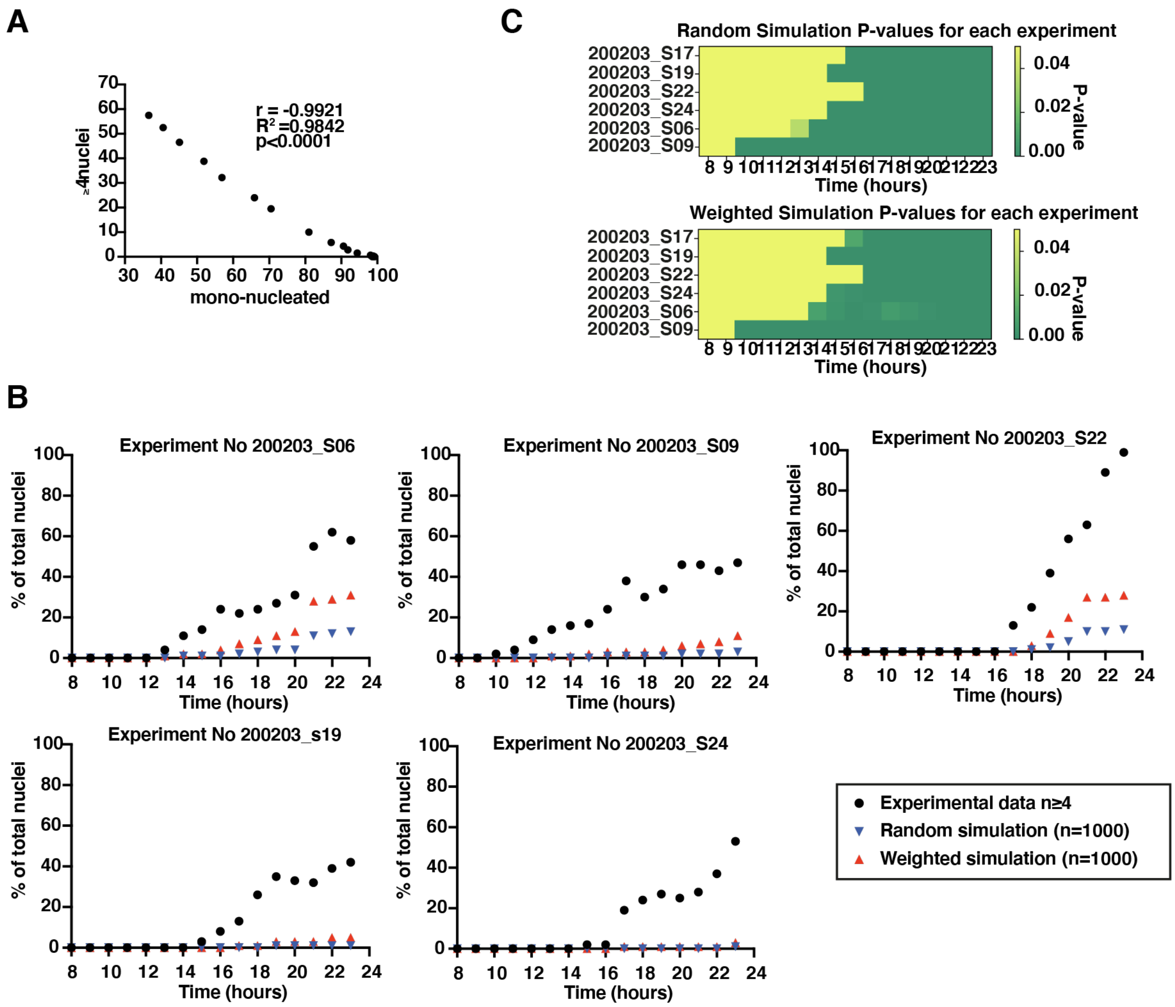

Supplemental Figure 2:

(**A**) Inverse correlation of mono-nucleated cells and multinucleated cells (≥ 4) from **Figure 2A**. (**B)** Data-driven simulations for five additional experiments establish that the fraction of nuclei present in multinucleated cells cannot be explained by random or weighted probabilities. See materials and methods and Figure 2C for details. (**C**) Corresponding p-values calculated with a bootstrapping approach for the fraction of nuclei in multinucleated cells for each time point in the experiment versus random (Top) and weighted simulations (Bottom). Significance level of 0.05 or lower was achieved after 10-17 hours from the onset of the experiment.

Supplemental Figure 3: Myoblast differentiation upon ERKi is not affected by blockade of calcium availability, related to Figure 3

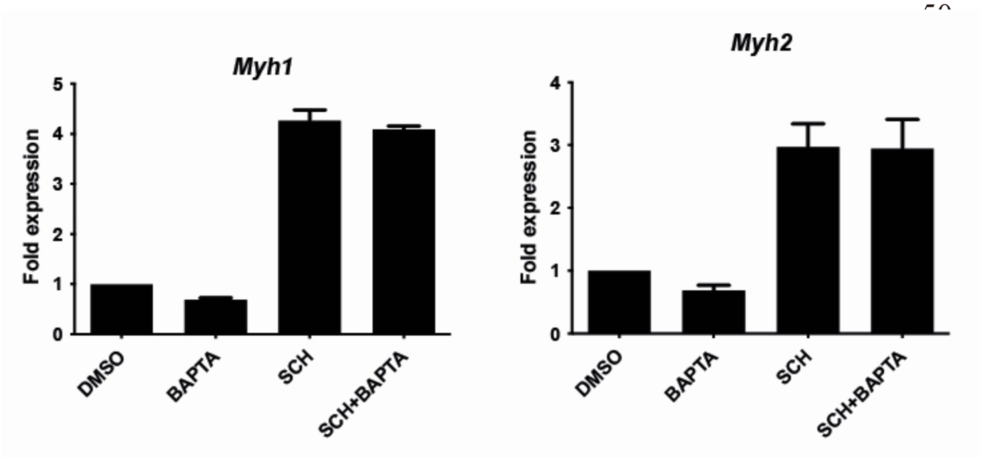

Supplemental Figure 3

Quantitative Realtime PCR analysis of the fold change in gene expression of myosin heavy chain 1 (Myh1) and 2 (Myh2) at 24 hours after treatment with either control (DMSO), 10uM BAPTA-AM, 1uM ERKi, or the combination of both ERKi and BAPTA-AM. Data is represents the mean +/- SEM of 3 biological repeats.

Supplemental figure 4. Inhibition of RXR and RYR inhibits fusion of DM treated myoblasts, related to Figure 3

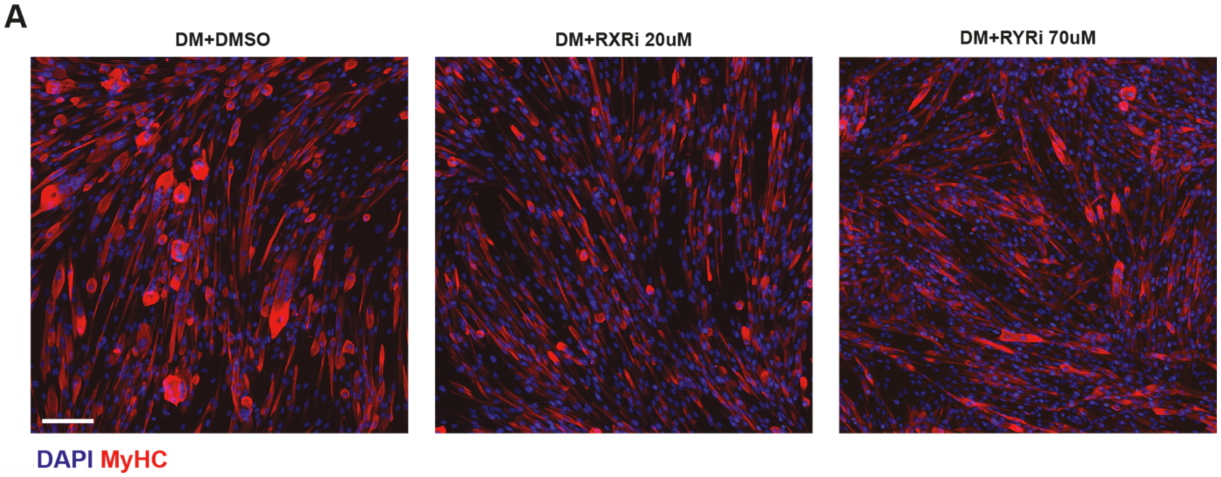

Supplemental figure 4:

(**A**) Representative IF staining of MyHC (red) and nuclei (DAPI, blue) for DMSO (ctrl), RXRi, or RYRi treated myoblasts grown in DM for 48 hours, showing their effect on fusion independent of ERKi. Scale bars, 100 μm.

Supplemental Figure 5: Evaluation of CaMKII activity downstream to ERKi, and its requirement for fusion, related to Figure 4

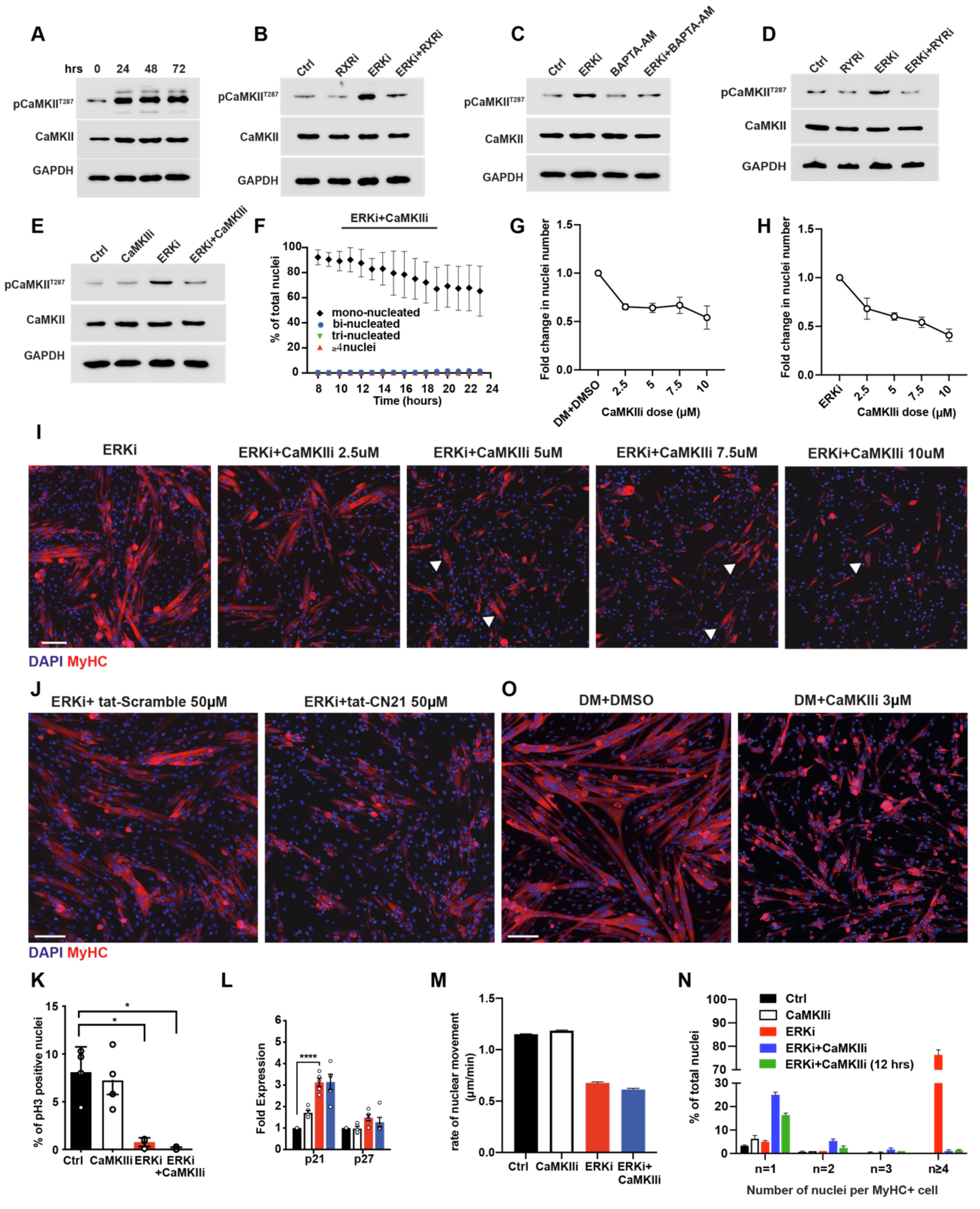

Supplemental Figure 5:

(**A**) Representative WB showing the activation of CaMKII in myoblasts treated with DM over 72 hours. (**B**) Representative WB showing the activation of CaMKII in myoblasts, treated with DMSO (Ctrl), 1 μM ERKi, 20 μM HX531(RXRi), or cotreated with ERKi and RXRi. (**C**) Representative WB showing the activation of CaMKII in myoblasts, treated with DMSO (Ctrl), 1 μM ERKi, 10μm BAPTA-AM, or cotreated with ERKi and BAPTA-AM. (**D**) Representative WBs showing CaMKII activation in myoblasts following 24 hours treatment with DMSO (Ctrl), 1 μM ERKi, Dantrolene 50 μM (RYRi), or cotreated with ERKi and RYRi. (**E**) Representative WB showing CaMKII activation of myoblasts treated with DMSO (Ctrl), 1 μM SCH772984 (ERKi), KN93 5 μM (CaMKIIi), or co-treated with ERKi and CaMKIIi at 24hrs post treatment. (**F**) Hourly fusion index following co-treatment of ERKi and CaMKIIi, showing the distribution of mono-, bi-, tri- and multi- nucleated (n≥4) cells. Total number of cells analyzed n=12,325. (**G**) Dose response of CaMKIIi treatment compared to treatment with DMSO, reported as change in nuclei number per field (**H**) Dose response of co-treatment with CaMKIIi compared to ERKi treatment alone, reported as change in nuclei number per field. (**I**) Representative IF images of MyHC (red) and nuclei (DAPI, blue) staining for the co-treatment dose response of CaMKIIi together with ERKi (1μm) at 24 hours post treatment. Arrows indicate bi or tri-nucleated cells that were still able to form. (**J**) Representative IF images of myoblasts co-treated with ERKi together with scrambled peptide (TAT-Scramble) or CaMKII peptide inhibitor (TAT-CN21) at 24 hours post treatment, MyHC (red) and nuclei (DAPI, blue). (**K**) Quantification of pH3 positivity following treatment with DMSO (Ctrl), 1 μM SCH772984 (ERKi), KN93 5 μM (CaMKIIi), or cotreated with ERKi and CaMKIIi at 24hrs post treatment (**L**) qRT-PCR expression analysis of *p21* and *p27* following co-treatment with ERKi and CaMKII, normalized to *Hprt*. Values are

expressed as fold change from that of DMSO (Ctrl). (**M**) Quantification of cell motility of myoblasts treated with DMSO (Ctrl), 1 μM SCH772984 (ERKi), KN93 5 μM (CaMKIIi), or cotreated with ERKi and CaMKIIi over a 24-hour period. (**N**) Stratified fusion index of cells which received a delayed co-treatment of CaMKII 12 hour following initial treatment with ERKi. (**O**) Representative IF images of myoblasts grown in DM or co-treated with CaMKIIi for 48 hours. All scale bars, 100 μm.

Supplemental Figure 6: RYR is exclusively localized to post-fusion myotubes and not to mononucleated cells, related to Figure 4

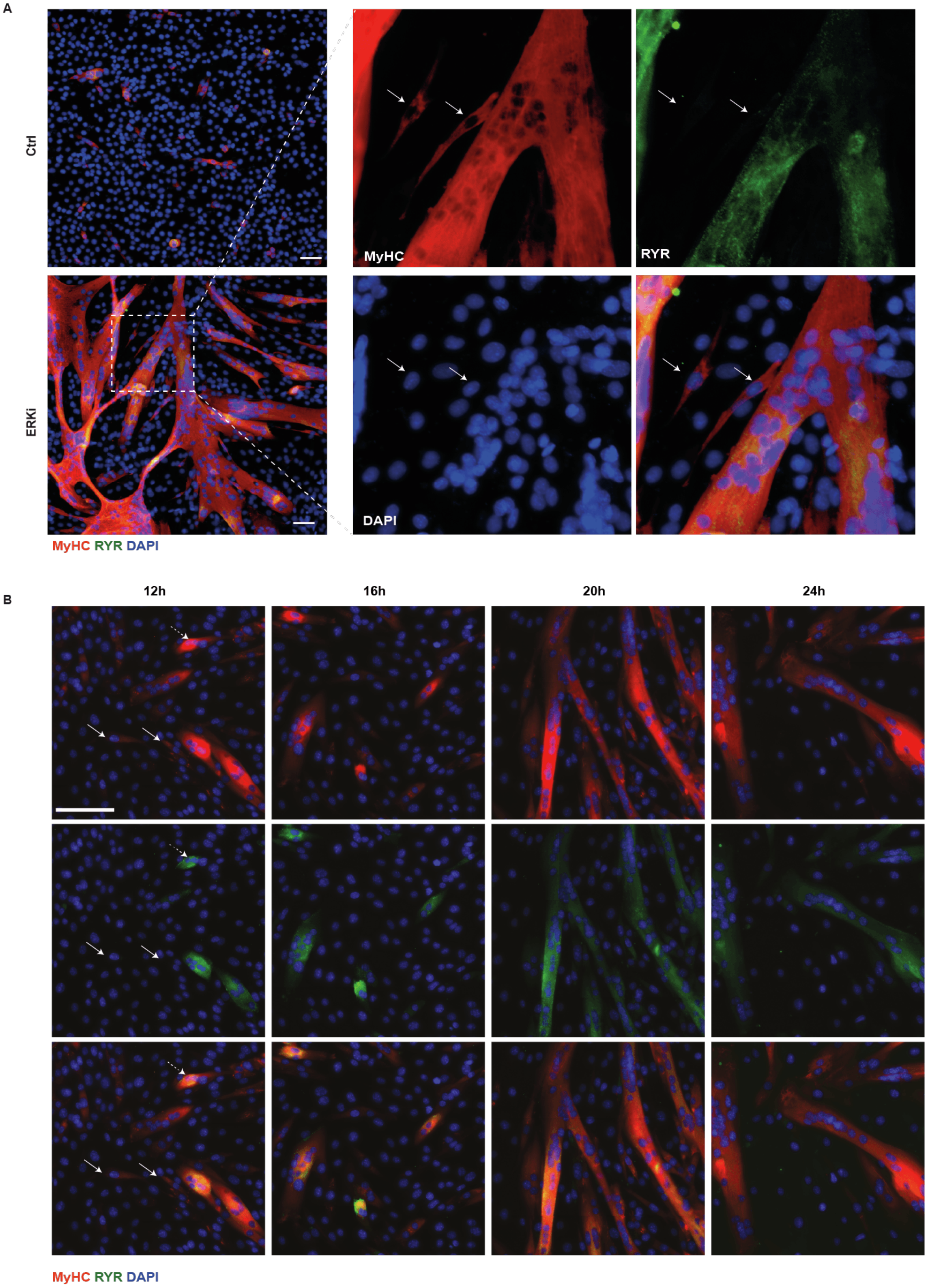

Supplemental Figure 6:

(**A**) Representative images of Ryanodine receptor (RYR) IF showing its localization in myotubes in Ctrl and ERKi treated cultures at 24hrs post-treatment. Indicated region in ERKi image is enlarged on right, showing the individual fluorescence channels and an overlay. Arrows indicate differentiated ($MyHC^{+}$) myoblasts lacking ryanodine receptor. (**B**) Representative IF images of RYR and MyHC at different time points after ERKi treatment showing that RYR is expressed in $MyHC^{+}$ multinucleated cells and mono nucleated cells (solid arrows). Dashed arrow indicates a bi-nucleated cell which is RYR positive. RYR (green), MyHC (red) and nuclei (DAPI, blue). Scale bars, 100 μm.

Supplemental Figure 7: Evaluation of successful PLA reactions, related to Figure 5

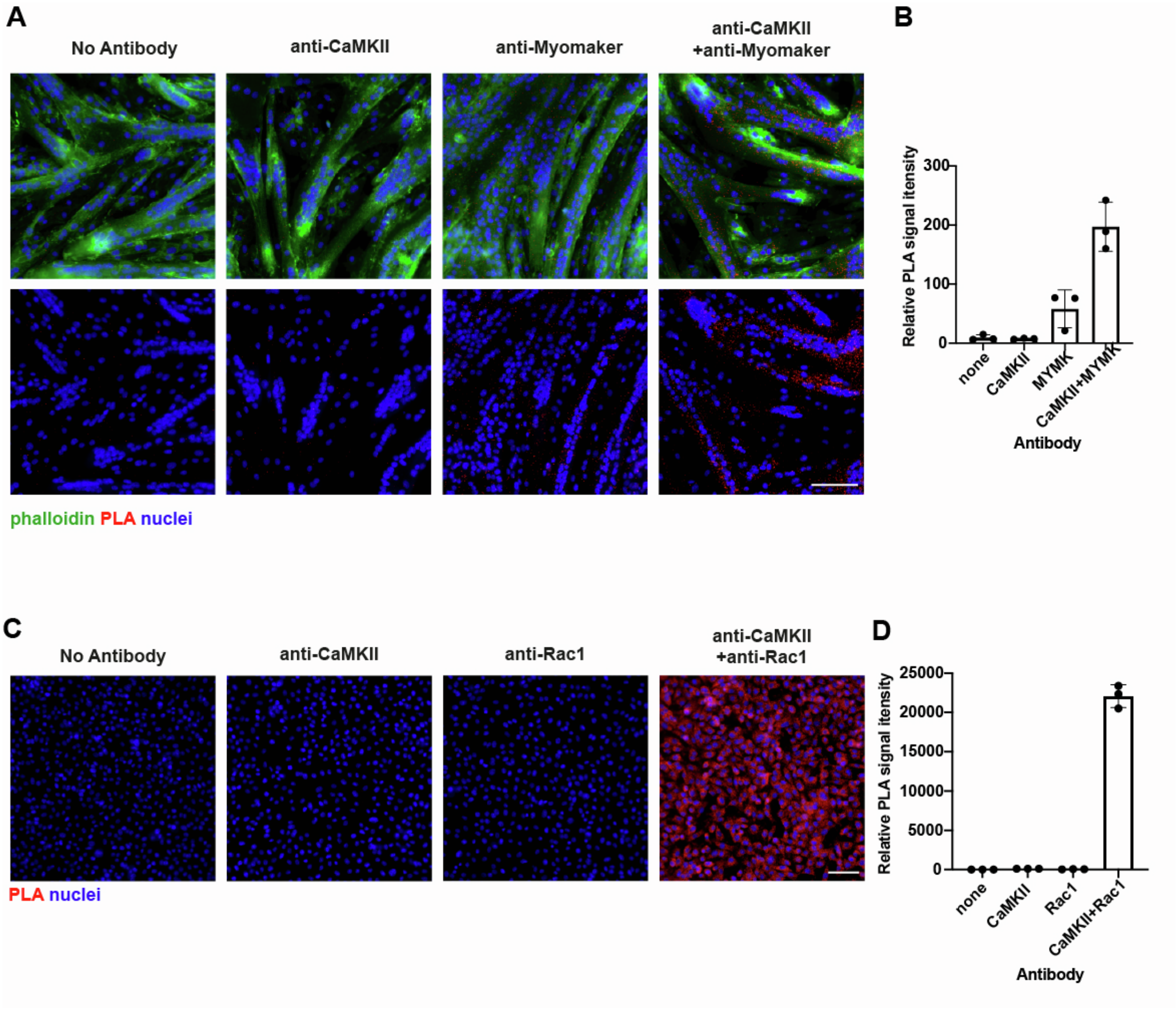

Supplemental Figure 7:

A) Negative controls for PLA interaction of Myomaker and CaMKII in ERKi induced myotubes. Top panel: Overlay of PLA signal (red), phalloidin (green) and DAPI (blue). Bottom panel: Overlay of the PLA signal (red) and DAPI (blue). Reactions were carried out either with no antibody or with the individual antibodies against Myomaker and CaMKII (anti-MYMK or anti-CaMKII respectively), or in comparison to the combination of both antibodies demonstrating the specificity of the PLA signal only when both antibodies are applied together. **B**) Quantification of the relative signal intensity of the PLA signal for each condition in **A**. The data represents the mean +/- SEM of 3 biological repeats. **C**) Negative controls for PLA interaction of Rac1 and CaMKII in proliferating myoblasts. Shown are the overlay of the PLA signal (red) and DAPI (blue). PLA reactions were carried out either with no antibody or with the individual antibodies against Rac1 or CaMKII (anti-Rac1 or anti-CaMKII respectively), or in comparison to the combination of both antibodies demonstrating the specificity of the PLA signal only when both antibodies are applied together. **D**) Quantification of the relative signal intensity of the PLA signal for each condition in **C**. The data represents the mean +/- SEM of 3 biological repeats. Scale bars = 100 μm.

Table 1: Primer list for qRT-PCR, related to Figure 1, 3, and 4

Gene	Forward Primer 5'→ 3'	Reverse Primer 5'→ 3'
Ryr1	ACGGAGAGAAAGTCATGGCG	ACTGATGGATTCCTGCAGCC
Ryr3	ACCAGCAGGAGCAAGTACG	GGGGTCGTGTCAAGTAGTCA
Orai1	GATCGGCCAGAGTTACTCCG	TGGGTAGTCATGGTCTGTGTC
Orai2	GACAGTCAGGCCTGGTCCC	CGGACCCAGTCTCGGTAATC
Stim1	CTTGCCCTGTGGCTTCTTTG	ATTCGGCAAAACTCTGCTTCG
Stim2	CTTGCGAGAACGGCTTTTTCG	GTACAGAGAGGAGGTGAGACTG
Itpr1	GGGTCCTGCTCCACTTGAC	CCACATCTTGGCTAGTAACCAG
Itpr2	TTCAGTTCCTATCGAGAGGATGT	GCTGATTGACGCAAGGTCG
Itpr3	GGGCGCAGAACAACGAGAT	GAAGTTTTGCAGGTCACGGTT
Atp2a1	TGTTTGTCCTATTTCGGGGTG	AATCCGCACAAGCAGGTCTTC
Atp2a2	TGGAACAACCCGGTAAGAGT	CACCAGGGGCATAATGAGCAG
Atp2a3	CGTCGCTTCTCGGTGACAG	AAGAGGTCCTCAAACTGCTCC
Pax7	CGGGTTCTGATTCCACATCT	CGACGAGGAAGGAGACAAGA
Myf5	ACGGCATGCCTGAATGTAAC	AGCTGGACACGGAGCTTTTA
Myog	GAAGCGCAGGCTCAAGAAAG	GCCGCGAGCAAATGATCTCC
MyoD	AACTGCTCTGATGGCATGATG	TGGAGATGCGCTCCACTATG
p27	CAGACGTAAACAGCTCCGAATTA	TCAGTGCTTATACAGGATGTCCA
p21	AGAGACAACGGCACACTTTG	CGGTGTCAGAGTCTAGGGGA
Mymk	GGGCTGTTCCATAGATGCTG	GGAGGCCATGGTCTACCTCT
Mymx	GTTAGAACTGGTGAGCAGGAG	CCATCGGGAGCAATGGAA
Gapdh	GGGTCCCAGCTTAGGTTCAT	CCAATACGGCCAAATCCGTT
Hprt	AGCGTCGTGATTAGCGATGA	GCAAGTCTTTCAGTCCTGTCC

Table 2: Primer list for cloning, related to Figure 2, 4, and 5

Cloning primer name:	Primer sequence 5'-->3'
CAMK2D-F	ATGGCTTCGACCACCACCT
CAMK2D-R	TTAGTTGATGGGTACTGTGG
XhoI-FLAG-CAMK2D-F	AGATCTCGAGCTCAAGATTACAAGGATGACGACGATAAGATGGCTTCGACCACCACCTGC
CAMK2D-T287V-IN-R:	AAGCAGTCTACATCCTCCTGCCTG
CAMK2D-BamHI-R	GGTGGATCCTCAGATGTTTTGCCACAAAGAGGT
CFP-F	GGCTTTTTTGGAGGCCTAGGCTTTTGCAAAAAGCTTACCATGGTGAGCAAGGGCGAG
CFP-R	CGGGTCGTGGGGCGGGCGTTATACCTTTCTCTTCTTTTTTGGATCTACCTT
dsRED-F	TGAGGAGGCTTTTTTGGAGGCCTAGGCTTTTGCAAAAAGCTTACCATGGCCTCCTCCGAGAACG
dsRED-R	ACTGACACACATTCCACAGGGTCGACCTCAGACACAAGTGCAGCA
MYMK-F	GCCCTCACTCCTTCTCTAGGCGCCGATGGGGACAGTTGTAGCCA
MYMK-R	ACTGACACACATTCCACAGGGTCGACCTCAGACACAAGTGCAGCA
