# Supplementary material for: ERK1/2 inhibition promotes robust myotube growth via CaMKII activation resulting in myoblast-to-myotube fusion
Source: Dev Cell. 2021 Dec 20;56(24):3349–3363.e6. doi: 10.1016/j.devcel.2021.11.022 (PMC8693863; doi:10.1016/j.devcel.2021.11.022)
Supplement: Document S2. Article plus supplemental information [file mmc8.pdf]

Developmental Cell

Article

ERK1/2 inhibition promotes robust myotube growth via CaMKII activation resulting in myoblast-to-myotube fusion

Graphical abstract

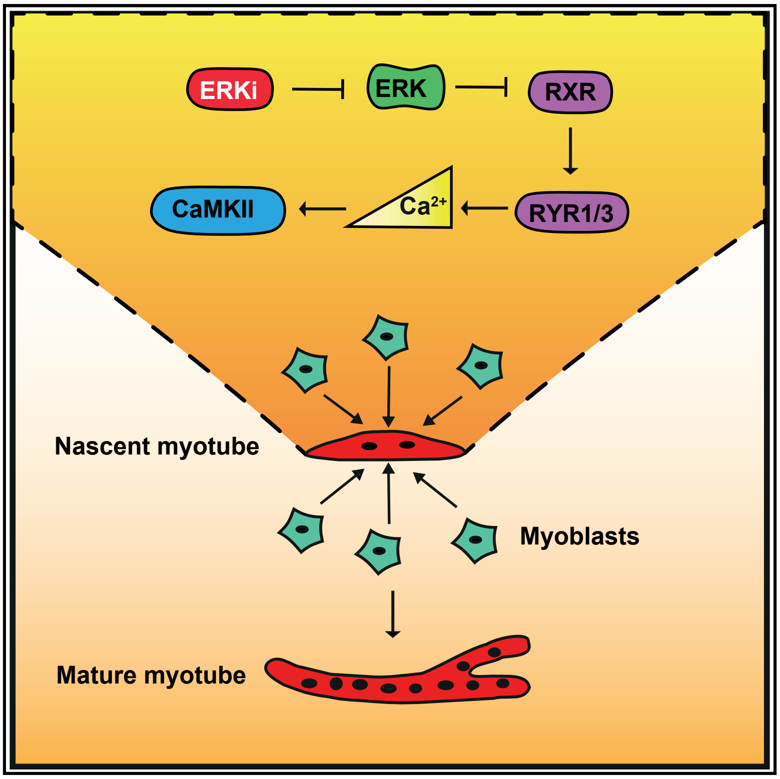

Highlights

- ERK inhibition induces robust mouse and chicken myoblast differentiation and fusion
- Myotubes fuse with mononucleated myoblasts at an asymmetric fusogenic synapse
- ERKi-driven signaling cascade leads to Ca^{2+}-CaMKII-dependent myoblasts to myotube fusion
- CaMKII is required for efficient muscle regeneration following injury

Authors

Tamar Eigler, Giulia Zarfati, Emmanuel Amzallag, ..., Douglas P. Millay, Eldad Tzahor, Ori Avinoam

Correspondence

eldad.tzahor@weizmann.ac.il (E.T.), ori.avinoam@weizmann.ac.il (O.A.)

In brief

Eigler et al. show that an evolutionarily conserved signaling cascade initiated by ERK inhibition in myoblasts leads to CaMKII-dependent fusion of mononucleated myoblasts with early myotubes at a fusogenic synapse. Moreover, CaMKII is required for efficient muscle regeneration following injury.

Eigler et al., 2021, Developmental Cell *56*, 3349–3363
December 20, 2021 © 2021 The Author(s). Published by Elsevier Inc.
https://doi.org/10.1016/j.devcel.2021.11.022

Developmental Cell

CellPress
OPEN ACCESS

Article

ERK1/2 inhibition promotes robust myotube growth via CaMKII activation resulting in myoblast-to-myotube fusion

Tamar Eigler,[1] Giulia Zarfati,[2] Emmanuel Amzallag,[1] Sansrity Sinha,[2] Nadav Segev,[2] Yishaia Zabary,[3] Assaf Zaritsky,[3] Avraham Shakked,[1] Kfir-Baruch Umansky,[1] Eyal D. Schejter,[4] Douglas P. Millay,[5,6] Eldad Tzahor,[1,7,*] and Ori Avinoam[2,*]

[1]Department of Molecular Cell Biology, Weizmann Institute of Science, Rehovot, Israel
[2]Department of Biomolecular Sciences, Weizmann Institute of Science, Rehovot, Israel
[3]Department of Software & Information Systems Engineering, Ben Gurion University, Be'er Sheva, Israel
[4]Department of Molecular Genetics, Weizmann Institute of Science, Rehovot, Israel
[5]Division of Molecular Cardiovascular Biology, Cincinnati Children's Hospital Medical Center, Cincinnati, OH, USA
[6]Department of Pediatrics, University of Cincinnati College of Medicine, Cincinnati, OH, USA
[7]Lead contact
*Correspondence: eldad.tzahor@weizmann.ac.il (E.T.), ori.avinoam@weizmann.ac.il (O.A.)
https://doi.org/10.1016/j.devcel.2021.11.022

SUMMARY

Myoblast fusion is essential for muscle development and regeneration. Yet, it remains poorly understood how mononucleated myoblasts fuse with preexisting fibers. We demonstrate that ERK1/2 inhibition (ERKi) induces robust differentiation and fusion of primary mouse myoblasts through a linear pathway involving RXR, ryanodine receptors, and calcium-dependent activation of CaMKII in nascent myotubes. CaMKII activation results in myotube growth via fusion with mononucleated myoblasts at a fusogenic synapse. Mechanistically, CaMKII interacts with and regulates MYMK and Rac1, and CaMKIIδ/γ knockout mice exhibit smaller regenerated myofibers following injury. In addition, the expression of a dominant negative CaMKII inhibits the formation of large multinucleated myotubes. Finally, we demonstrate the evolutionary conservation of the pathway in chicken myoblasts. We conclude that ERK1/2 represses a signaling cascade leading to CaMKII-mediated fusion of myoblasts to myotubes, providing an attractive target for the cultivated meat industry and regenerative medicine.

INTRODUCTION

During embryonic muscle development, myoblasts proliferate and undergo terminal differentiation, a multistep process which requires cell-cycle withdrawal, initiation of a muscle-specific gene transcriptional program, differentiation into fusion-competent myoblasts, and ultimately cell-to-cell fusion to form nascent multinucleated myotubes that mature to form contractile muscle fibers (Chal and Pourquié, 2017; Dumont and Rudnicki, 2017; Hernández-Hernández et al., 2017; Schmidt et al., 2019). This process is recapitulated during muscle regeneration due to the presence of satellite cells (SCs), the resident muscle stem cell. (Chal and Pourquié, 2017; Dumont and Rudnicki, 2017; Hindi et al., 2013). However, defining the molecular signaling pathways that specifically regulate cell-to-cell fusion remain challenging owing to the difficulty in distinguishing processes that regulate fusion from those that regulate myogenic differentiation, which will inevitably, although indirectly, affect fusion.

The study of *Drosophila* muscle development has highlighted many facets of myoblast fusion, particularly the critical role of cytoskeletal rearrangement and the formation of membrane protrusions that extend from an "advancing" myoblast to a "receiving" myotube (Chen, 2011; Kim and Chen, 2019; Kim et al., 2015; Lee and Chen, 2019; Lehka and Rędowicz, 2020; Schejter, 2016; Shilagardi et al., 2013). *Drosophila* muscle development has been described as a two-phase process. The first phase leads to the formation of founder cells, small nascent myotubes consisting of 2–3 nuclei (Beckett and Baylies, 2007; Önel and Renkawitz-Pohl, 2009; Rau et al., 2001). Founder cells attract surrounding fusion-competent myoblasts and fuse with them to form large multinucleated myotubes that mature into muscle fibers (Abmayr and Pavlath, 2012; Chen and Olson, 2005; Hernández and Podbilewicz, 2017; Rochlin et al., 2010; Schejter, 2016; Segal et al., 2016). Despite many conserved similarities between *Drosophila* and vertebrate muscle fusion, differences do exist. For example, Myomaker (*Mymk*, a.k.a TMEM8c) and Myomixer (*Mymx*, a.k.a GM7325, Myomerger, or Minion), two muscle-specific proteins which were shown to be essential and sufficient for myoblast fusion in vertebrates, are absent in invertebrates (Leikina et al., 2018; Millay et al., 2016, 2013, 2014; Quinn et al., 2017). Moreover, it is unclear whether the biphasic phenomenon of myotube growth described in *Drosophila* is conserved in vertebrate muscle fusion, and if so, whether the processes that regulate myoblast-to-myoblast fusion (primary

This is an open access article under the CC BY license (http://creativecommons.org/licenses/by/4.0/).

fusion) and myoblast-to-myotube fusion (secondary fusion) are distinct.

The mitogen-activated protein kinases (MAPKs), including p38, JNK, ERK1/2, and ERK5, mediate diverse signaling pathways, and are all implicated in muscle development and myoblast differentiation (Alter et al., 2008; Knight and Kothary, 2011; Segalés et al., 2016; Xie et al., 2018). However, the role of ERK1/2 in muscle fusion remains unclear and largely contradictory (Bennett and Tonks, 1997; Dinev et al., 2001; Jones et al., 2001; Sarbassov and Peterson, 1998; Sarbassov et al., 1997; Shi et al., 2018; Sunadome et al., 2011; Tiffin et al., 2004; Wu et al., 2000; Yang et al., 2006). ERK1/2 promotes myoblast proliferation in response to various growth factors (Campbell et al., 1995; Scata et al., 1999); inhibition of signaling pathways leading to ERK1/2 activation or sequestering ERK1/2 in the cytoplasm results in cell-cycle exit and differentiation (Jones et al., 2001; Michailovici et al., 2014; Sarbassov et al., 1997; Tiffin et al., 2004; Wu et al., 2000). In cancer cell lines, ERK1/2 phosphorylates the nuclear retinoid-X receptor (RXR), leading to inhibition of its transactivation potential (Macoritto et al., 2008; Matsushima-Nishiwaki et al., 2001), and RXR activity in myoblasts promotes myogenesis through regulation of *MyoD* expression and as a MYOG co-factor (Alric et al., 1998; Froeschlé et al., 1998; Khilji et al., 2020; Le May et al., 2011; Zhu et al., 2009).

Calcium (Ca^{2+}) has long been implicated as a regulator of mammalian muscle fusion (Constantin et al., 1996; Shainberg et al., 1969). Transient Ca^{2+} depletion from the endoplasmic reticulum (ER) is associated with myoblast differentiation and fusion (Nakanishi et al., 2015). Moreover, the Ca^{2+}-sensitive transcription factor, NFATc2, was reported to mediate myoblast recruitment and myotube expansion (Horsley et al., 2003). Yet the signaling cascades which lead to Ca^{2+}-mediated myoblast fusion remain unclear. Intracellular Ca^{2+} levels are regulated through various Ca^{2+} and voltage-gated channels, including but not limited to ryanodine receptors (RYRs). RYRs are Ca^{2+} channels expressed on the ER, which regulate Ca^{2+} efflux into the cytosol. RYRs were previously implicated in the regulation of muscle terminal differentiation, but not myogenic commitment in fetal myoblast differentiation (Pisaniello et al., 2003).

CaMKII is a member of the Ca^{2+}/calmodulin (CaM)-dependent serine/threonine kinase family. CaMKII delta (δ) and gamma (γ), and to some extent beta (β), are the primary isoforms expressed in skeletal muscle (Bayer et al., 1996). Upon Ca^{2+}/CaM binding to individual CaMKII subunits, cross-phosphorylation of neighboring subunits at T287 leads to a state of autonomous activation, by increasing the affinity for Ca^{2+}/CaM several thousand-fold. Previously, CaMKII was identified for its role in Ca^{2+}-dependent regulation of gene expression associated with muscle-oxidative metabolism, as well as components of the contractile machinery (Eilers, 2014a; Eilers, 2014b; Moradi, 2020; Ojuka, 2012; Richter and Hargreaves, 2013; Rose, 2007). However, to date, the specific role of CaMKII in the regulation of myoblast fusion has not been demonstrated.

By using the highly specific ERK1/2 inhibitor SCH772984 (Morris et al., 2013) in primary mouse and chick myoblast cultures, we describe here the pleiotropic role of ERK1/2 during myogenesis. First, in the inhibition of cell-cycle exit and initiation of the myogenic transcriptional program, and second in the suppression of a signaling cascade that culminates in CaMKII-dependent regulation secondary myoblast-to-myotube fusion. Moreover, we demonstrate a requirement for CaMKII during muscle regeneration.

RESULTS

ERK1/2 inhibition (ERKi) induces myoblast differentiation and hyperfusion in proliferation medium

Based on the recent findings by us and others, we hypothesized that ERK1/2 prevents myogenesis not only through maintenance of myoblast proliferation but also through the active repression of pro-myogenic nuclear targets (Michailovici et al., 2014; Yohe, 2018). In order to examine the role of ERK1/2 in myoblast differentiation and fusion, early-passage mouse-derived primary myoblasts were treated with the ERK1/2 inhibitor SCH772984 (ERKi, 1 μM) while in proliferation medium (PM). SCH772984 is a highly selective, ATP-competitive inhibitor of both ERK1 and ERK2. It acts by directly effecting ERK kinase activity and simultaneously inhibiting MEK-mediated phosphorylation of ERK through allosteric mechanisms (Morris et al., 2013; Nissan et al., 2013). ERKi resulted in the robust formation of myotubes (Figures 1A and 1B; Video S1) as compared with conventional serum-reduced differentiation medium (DM) (90.5% in ERKi versus 11.6% in DM after 24 h). The differentiation and fusion factors *MyoD, MyoG*, *Mymk*, and *Mymx* were upregulated much earlier in cells treated with ERKi compared with DM alone (Figure 1C). In addition, the fraction of $MYOG^{+}$ nuclei was significantly higher for ERKi compared with DM alone (Figures 1D and 1E). Moreover, immunofluorescence staining of ERKi cultures with the proliferation markers KI-67 (Figures 1F and 1G) and phosphorylated histone 3 (pH3) (Figures 1H and 1I) demonstrated that myoblasts undergo cell-cycle arrest, consistent with differentiation. ERKi also induced a similar effect on myoblasts cultured in DM (Figures S1A–S1C). Taken together, these results show that ERKi induces a more robust differentiation and fusion response in PM and in DM as compared with myoblasts cultured in DM alone, suggesting that ERK1/2 acts as a repressor of cell-cycle exit and initiation of the myogenic transcriptional program.

Myotubes grow through recruitment of mononucleated myoblasts at a fusogenic synapse

As we observed that myoblasts treated with ERKi exhibited a more robust fusion phenotype compared with cells in conventional DM, we wondered if ERKi was activating processes leading to increased myotube expansion through secondary fusion of mononucleated myoblasts and myotubes, as previously described in *Drosophila* (Önel and Renkawitz-Pohl, 2009). To explore this, we performed live-cell imaging of myoblasts expressing a membrane-targeted GFP and cytoplasmic DsRed and calculated an hourly fusion index for a period of 8–23 h post ERKi. We found that after the initial formation of bi- and trinucleated cells, these cells accumulated nuclei and expanded rapidly through several fusion events with mononucleated cells (Figures 2A, 2B, and S2A; Videos S1 and S2).

The observed expansion of myotubes at the expense of mononucleated cells is either a regulated phenomenon or a stochastic process, wherein the larger multinucleated myotubes grow

CellPress
OPEN ACCESS

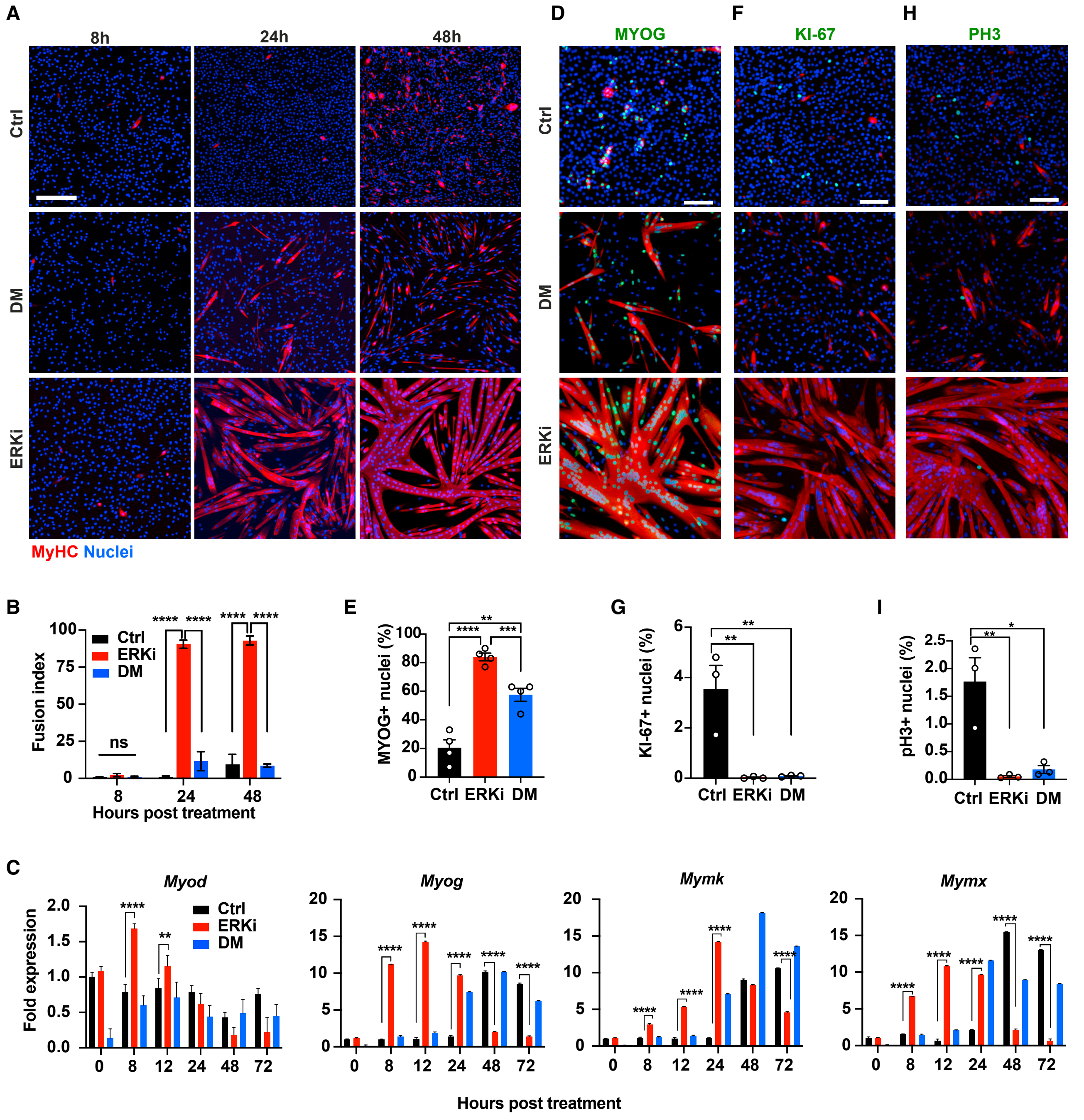

Figure 1. ERK1/2 inhibition induces myoblast differentiation and hyperfusion in proliferation medium

(A) Representative immunofluorescent (IF) images of myoblasts at 8, 24, and 48 h after treatment with DMSO (Ctrl) or 1 μM SCH772984 (ERKi) in proliferation medium (PM) or in differentiation medium (DM). Cells were stained with myosin heavy chain (MyHC, red), and the nuclear dye Hoechst (blue). Scale bar: 200 μm.

(B) Fusion index of (A) representing the percent of total nuclei found in $MyHC^{+}$ cells with two or more nuclei (total nuclei assayed, n = 88,518).

(C) Representative qRT-PCR results showing the temporal gene-expression profiles of *Myod*, *Myog*, *Mymk*, and *Mymx*, normalized to *Gapdh*, during myogenesis. Values are expressed as fold change from the control at 0 h.

(D, F, and H) Representative images of myoblasts treated with DMSO (Ctrl) or 1μM ERKi in PM or DM for 24 h and stained for MyHC (red), and MYOG (green) (D); MyHC (red) and Ki-67 (green) (F); and MyHC (red) and pH3 (green) (H). Nuclei are stained with DAPI (blue). Scale bar: 100 μm.

(E, G, and I) Percentage of MYOG, Ki-67, and pH3 positive nuclei, respectively. All data are representative of at least 3 biological repeats. Error bars indicate SEM.

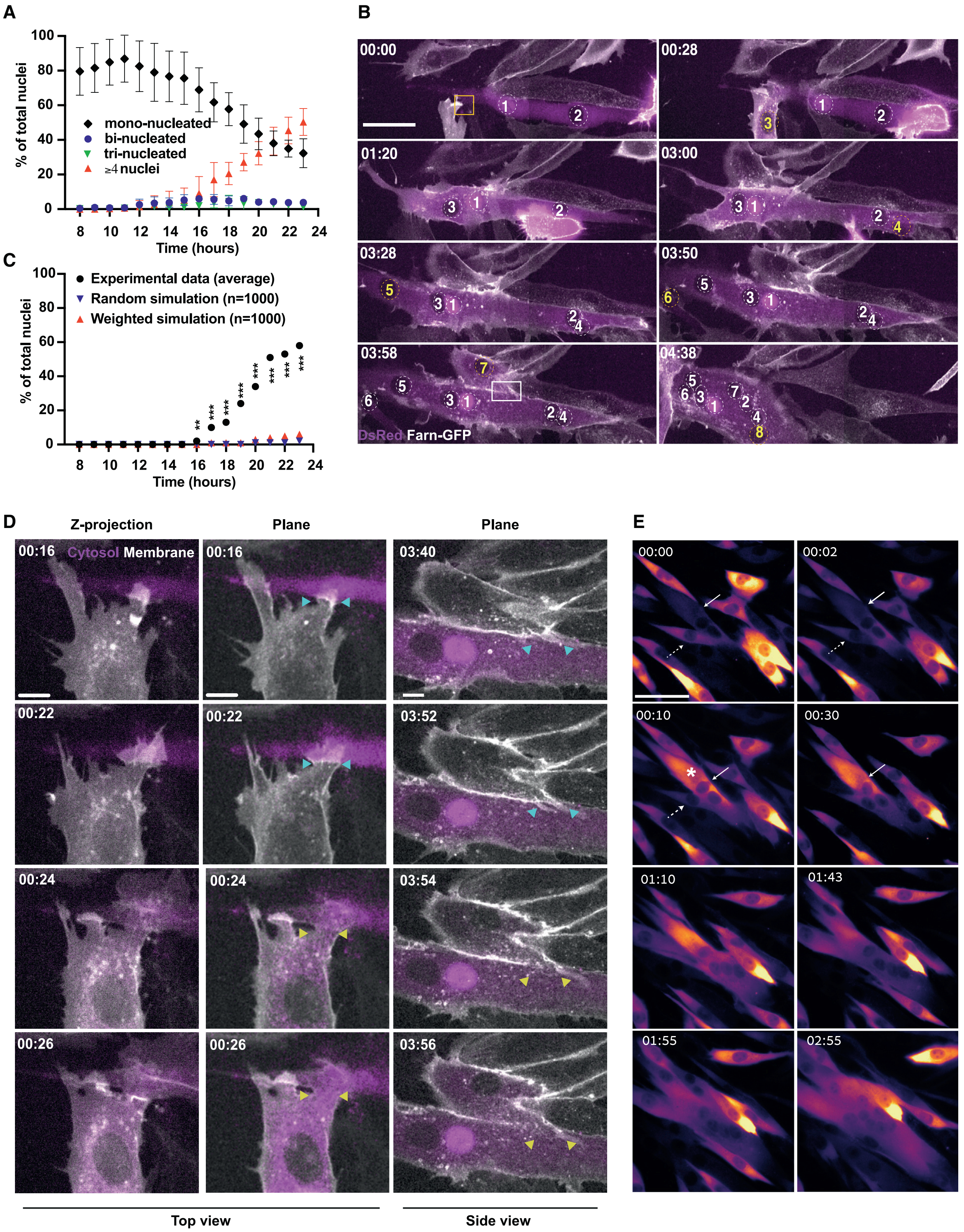

(legend on next page)

rapidly because of their inherent higher probability to interact and fuse with neighboring cells. To test this, we performed data-driven simulations (Figures 2C, S2B, and S2C). We considered two scenarios, one where all cells have an equal probability to fuse (random simulation) and one where the probability to fuse was dependent on cell size (weighted simulation). However, neither of the simulations recapitulated our results, implying that myotube growth is not stochastic in nature (Figures 2C, S2B, and S2C). Time-lapse microscopy also revealed that, starting at 8 h after ERKi treatment, myoblasts begin to display concerted collective movement and an increase in actin-rich membrane protrusions (Videos S1 and S3). Moreover, it showed that fusion occurs at a single location, where a protrusion extends from the advancing myoblast to the receiving myotube (observed in 85% of fusion events; n = 46) (Figure 2D; Video S4).

As Ca^{2+} has long been implicated in processes specifically associated with myoblast fusion, we visualized Ca^{2+} dynamics during ERKi-induced myogenesis by imaging myoblasts harvested from GCaMP6 Ca^{2+} reporter mice. We observed that a pulse of Ca^{2+} in nascent myotubes precedes the phase of rapid myotube growth, suggesting that Ca^{2+} released from the ER in early myotubes may facilitate secondary fusion and myotube expansion (Figure 2E; Video S5). Taken together, these results suggest that myotube growth in mammals is initiated by the generation of multinucleated founder cells (2–3 nuclei) that expand by fusion of "advancing" myoblasts to the "receiving" myotube, and that this process might be regulated by cytosolic Ca^{2+}.

ERK1/2 inhibition initiates an RXR/RYR-dependent fusion response

To better understand the role of cytosolic Ca^{2+} in secondary fusion, we examined the gene expression of various Ca^{2+} channels. Ryanodine receptors (RYR1-3) are channels that mediate the release of Ca^{2+} stores from the sarcoplasmic reticulum (SR) into the cytoplasm during excitation-contraction coupling in both cardiac and skeletal muscle cells. The expression of *Ryr1* and *Ryr3*, as well as Ca^{2+}-sensing channels such as SERCA1/2 (*Atp2a1* and *Atp2a2*), *Orai1*/2, and *STIM1*/2 were upregulated in ERKi-treated myoblast cultures (Figure 3A). Co-treatment of cultures with ERKi and the RYR-specific antagonist dantrolene (50 μM, RYRi) reduced fusion by 60% (Figures 3B and 3C) without affecting differentiation, measured by the fraction of $MYOG^+$ nuclei (Figures 3B and 3D). Along the same line, myoblasts co-treated with ERKi and the Ca^{2+} chelator BAPTA-AM (10 μM) exhibited reduced fusion by 81% (Figures 3B and 3E), without affecting myogenic differentiation (Figures 3B, 3F, and S3). Taken together, these results imply that elevated levels of cytosolic Ca^{2+} are essential for myoblast fusion.

As we previously reported, ERK1/2 nuclear localization represses myogenic differentiation, while sequestration of ERK in the cytoplasm promotes differentiation (Michailovici et al., 2014). We thus hypothesized that ERK1/2 may repress differentiation through the phosphoinhibition of a nuclear transcription factor. As RXR regulates myogenesis and is also shown to undergo phosphoinhibition at S260 by ERK1/2, we asked whether RXR might be a nuclear ERK1/2 target in proliferating myoblasts upstream of *Ryr1* and *Ryr3*. Co-treatment of myoblasts with ERKi and the specific RXR antagonist HX531 (20 μm, RXRi) resulted in the downregulation of *Ryr1* and *Ryr3* mRNA expression (Figure 3G). RXR immunoprecipitated with ERK1/2 in myoblasts grown in proliferation conditions, and this interaction was attenuated upon treatment with ERKi (Figure 3H). Co-treatment with RXRi similarly led to inhibition of fusion by 47% at 24 h after treatment (Figures 3B and 3I), without affecting differentiation, as measured by the fraction of $MYOG^+$ nuclei (Figures 3B and 3J). Consistently, treatment with RXRi and RYRi generated a similar reduction in fusion in cultures grown in DM (Figure S4). Moreover, time-course experiments demonstrated a reduction in phosphorylated RXR within 15 min of administration of ERKi, coinciding with the reduction of ERK1/2 phosphorylation (Figure 3K). These data imply that in proliferating myoblasts, RXR is directly regulated by ERK1/2 and that upon ERK inhibition, phosphoinhibition of RXR is relieved, leading to the transactivation of *Ryr1* and *Ryr3* expression, which likely promotes Ca^{2+} release from the ER, resulting in myoblast fusion with the growing myotube.

Myotube expansion requires calcium-dependent CaMKII activation

Next, we wondered if Ca^{2+}-dependent phosphorylation and activation of cellular kinases might be involved in regulating fiber growth through secondary fusion. We found that the Ca^{2+}-dependent enzyme CaMKII was activated by phosphorylation at the T287 residue upon treatment of myoblasts with ERKi in PM, as well as following treatment in DM for 24 h (Figures 4A and S5A). CaMKII activation begins at 12 h following ERKi treatment, coinciding with the increase in total and phosphorylated RYR protein levels and with the onset of myotube expansion by secondary

Figure 2. Myotubes grow through recruitment of mononucleated myoblasts at a fusogenic synapse

(A) Hourly fusion index showing the distribution of mono-, bi-, tri-, and multinucleated (n ≥ 4) cells. Total number of nuclei assayed, n = 13,044.

(B) Representative frames from time-lapse microscopy of an individual growing myotube (Video S2). At time 0 a binucleated myotube labeled with a cytoplasmic DsRed (purple) is approached by a mononucleated myoblast (yellow square) expressing a membrane-targeted GFP (white). When the cells fuse, cytoplasmic and membrane mixing become apparent (t = 00:28). Scale bar: 50 μm. Yellow and white squares mark the fusion events shown in (D).

(C) Experimental data compared with simulated data in two stochastic fusion scenarios: equal probability of cells to fuse irrespective of their number of nuclei (≥ 4 nuclei), and weighted probability, which considered the possibility that the probability of a cell to add nuclei was proportional to the number of nuclei within it (see STAR Methods for full details).

(D) Two examples of "fusogenic synapses" from the expanding fiber in (B) (time: hh:mm). Scale bar: 10 μm. Left column: Z-projection of the confocal stack. A protrusion extending from the myoblast to the myotube where fusion eventually occurs as can be seen by the simultaneous diffusion of the cytoplasmic marker into the myoblast and the disappearance of the membrane marker from the protrusion between the two fusing cells (Video S4). Middle and right columns: focal planes from two events where the fusion pore can be seen expanding. Cyan and yellow arrows point to the fusogenic synapses before and after fusion.

(E) Representative frames acquired of GCaMP6S Ca^{2+} reporter fluorescence in a growing myotube undergoing expansion via fusion (Video S5). Arrows indicate a myoblast and a small myotube before fusion and the initiation of fiber growth. Dashed arrow indicates a myoblast prior to and during fusion. Asterisk indicates burst in GCaMP6S fluorescence. Scale bar: 50 μm. Time in (B), (D), and (E) (hh:mm).

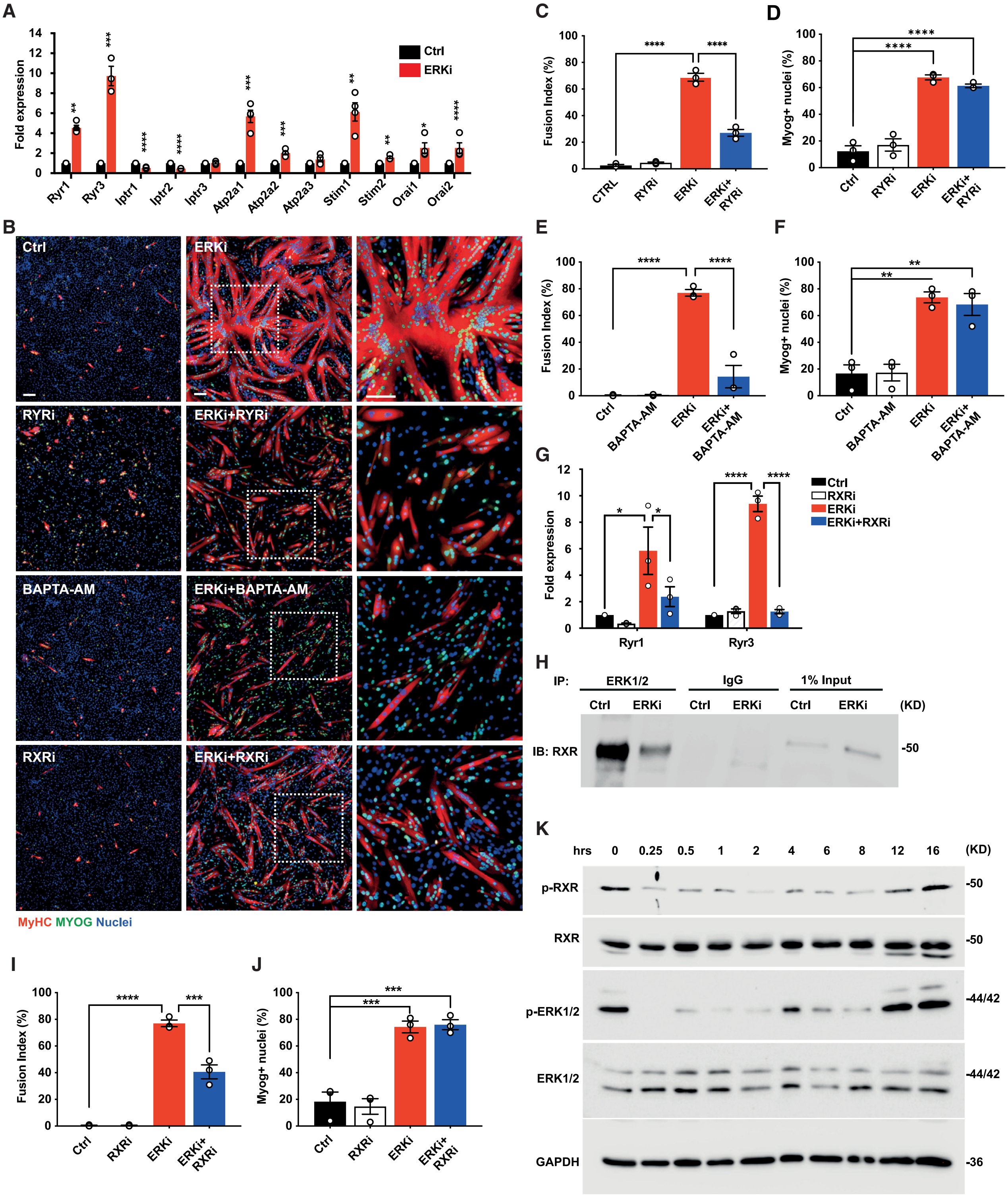

Figure 3. ERK1/2 inhibition initiates an RXR/RYR-dependent fusion response

(A) qRT-PCR analysis of fold change in expression of Ca^{2+} channels and sensors in DMSO (Ctrl) compared with ERKi-treated cells at 24 h; expression was normalized to *Hprt*.

(B) Representative IF images of cells treated with DMSO (Ctrl), 1 μM ERKi, 50 μM dantrolene (RYRi), ERKi, and RYRi, 10 μM BAPTA-AM, ERKi and BAPTA-AM, 20 μM HX531(RXRi), or ERKi and RXRi at 24 h. The differentiation markers MyHC (red), MYOG (green), and nuclei (blue) are shown. White boxes indicate the region enlarged on the right.

(legend continued on next page)

fusion (Figures 4B, 2A, and S6). Moreover, CaMKII activation following ERKi is dependent on the upstream activity of RYR, RXR, and Ca^{2+} (Figures S5B–S5D, respectively). Strikingly, co-treatment with the CaMKII inhibitor KN93 (5 μM; CaMKIIi) suppressed the formation of polynucleated myotubes but maintained bi- and trinucleated $MyHC^{+}$ cells, without affecting differentiation (Figures 4C–4F, S5E, and S5F). Bi- and trinucleated myotubes were still apparent even at higher concentrations of CaMKIIi, which began to show toxicity at 10 μM (Figures S5G–S5I). In addition, the CaMKII inhibitor tat-CN21 (a phosphomimetic peptide) gave a similar fusion suppression phenotype (Figure S5J). Co-treatment of ERKi with CaMKIIi did not affect cell-cycle arrest, as measured by pH3 staining (Figure S5K) or expression of the cell-cycle inhibitors *p21* and *p27*, compared with ERKi alone (Figure S5L), nor did it affect cell motility, demonstrating that fusion failure is not due to an effect on cell-cycle arrest or cell migration (Figure S5M; Video S6).

To further evaluate if the effect of CaMKII inhibition was specific to myotube growth through secondary fusion, we administered CaMKIIi at 12 h following treatment with ERKi, coinciding with the time point at which its activation was observed. Late addition of CaMKIIi resulted in a phenotype not significantly different from its addition at time 0, indicating that CaMKII inhibition has no effect before secondary fusion begins (Figure S5N). CaMKIIi also had a similar effect on myoblasts cultured in DM for 48 h, showing that the effect of CaMKIIi is not dependent on ERKi (Figure S5O). These results suggest that CaMKII activation is essential for myoblast-to-myotube fusion but not for myoblast-to-myoblast fusion. Therefore, in the presence of CaMKIIi, bi- and trinucleated myotubes form but fail to expand into large multinucleated fibers. Consistently, both RYR and phosphorylated CaMKII are primarily localized to myotubes rather than to mononucleated MyHC cells, following ERKi treatment (Figures S6A, S6B, and 4G, respectively).

To examine whether CaMKII activation is sufficient to induce myoblast-to-myotube fusion independent of treatment with ERKi, primary myoblasts were transduced with either empty adenovirus vector (Ad-Ctrl), wildtype CaMKII (Ad-CaMK2δ^{WT}), or phospho-null CaMKII (Ad-CAMK2δ^{T287V}), and induced to differentiate in DM. We found, as expected, that following treatment in DM for 72 h, exogenous CAMK2δ^{WT} was activated by phosphorylation, yet CAMK2δ^{T287V} failed to undergo activation (Figure 4H). Importantly, we observed that while expression of CAMK2δ^{WT} enhanced formation of bi- and polynucleated MyHC+ cells, expression of CAMK2δ^{T287V} did not; it rather suppressed growth of multinucleated cells compared with the control (Figure 4I). Taken together, the results suggest that CaMKII activation is sufficient to promote secondary (myoblast-to-myotube) fusion and implies a role for CaMKII function in myotubes.

CaMKII interacts with and regulates MYMK and Rac1 during fusion

The expression of both *Mymk* and *Mymx* was elevated upon treatment with ERKi (Figure 1C); However, the increase in *Mymk* expression, but not of *Mymx*, was partially suppressed upon co-treatment with ERKi and CaMKIIi (Figure 4F). Therefore, we examined whether reduced fusion upon CaMKII inhibition could be attributed to decreased *Mymk* expression. To assess this, we overexpressed MYMK by retroviral transduction in primary myoblasts and subjected them to treatment with ERKi and CaMKIIi. We found that ERKi-dependent fusion was enhanced upon overexpression of MYMK (Figures 5A and 5B). However, this effect was completely dependent on CaMKII activity as large myotubes were lost upon co-treatment with CaMKIIi, while the accumulation of mono-, bi-, and trinucleated cells was similar to that of cells transduced with control retrovirus (Figures 5A–5C).

These data suggested that CaMKII may interact with and regulate MYMK activity. To test this, we used a proximity ligation assay (PLA), which demonstrated that CaMKII and MYMK PLA signal mean fluorescent intensity was increased by 2.9-fold following ERK inhibition (Figures 5C, 5D, and S7). Moreover, the PLA signal was exclusive in myotubes and not in mononucleated cells, similar to the expression pattern for RYR and p-CaMKII (Figures 5D, 4G, S7A, and S7B, respectively). Due to the increase in actin-rich protrusions observed upon ERKi (Video S3), we briefly explored potential interactions of CaMKII with the actin reassembly machinery. Interestingly, the RhoGTPase Rac1, which is required for fusion, was predicted as an *in silico* CaMKII target at the serine 71 residue (Wang et al., 2020). Indeed, we show that increased Rac1 phosphorylation at S71 is dependent on CaMKII activity following ERK inhibition (Figures 5E, S7C, and S7D). Moreover, we demonstrated a significant increase in the PLA signal between CaMKII and Rac1 following ERK inhibition (Figures 5F and 5G). Taken together, these results suggest that Ca^{2+}-dependent CaMKII activation is a downstream event to the activation of RXR and RYR, and that CaMKII activity is essential in myotubes for their expansion by mediating myoblast-to-myotube fusion, likely through regulation of MYMK and Rac1.

CaMKII function during muscle regeneration and ERK-CaMKII pathway conservation

To examine the role of CaMKII during muscle regeneration *in vivo*, wild-type (WT) mice were subjected to cardiotoxin (CTX) induced injuries, and tissues were collected on the day of injection, and consecutively on days 2–8 post injury. We observed an acute activation of ERK1/2 two days post CTX injury, likely associated with increased proliferation of myoblasts

(C and D) Fusion index and quantification of percent of $MYOG^{+}$ nuclei, respectively, for the ERKi and RYRi co-treatment experiment. Total number of nuclei assayed, n = 113,448.

(E and F) Fusion index and quantification of percentage of $MYOG^{+}$ nuclei, respectively, for the ERKi and BAPTA-AM co-treatment experiment. Total number of nuclei assayed, n = 109,360.

(G) qRT-PCR analysis of *Ryr1/3* gene expression following co-treatment with ERKi and RXRi.

(H) Co-immunoprecipitation of ERK1/2 with RXR.

(I and J) Fusion index and quantification of percent of $MYOG^{+}$ nuclei, respectively, for the ERKi and RXRi co-treatment experiment. Total number of nuclei, n = 106,116.

(K) Representative western blot (WB) showing inhibition of ERK1/2 and reduction in phosphorylated RXR within 15 min post addition of ERKi. All data are representative of 3 biological repeats. Error Bars indicate SEM. Scale bars: 100 μm.

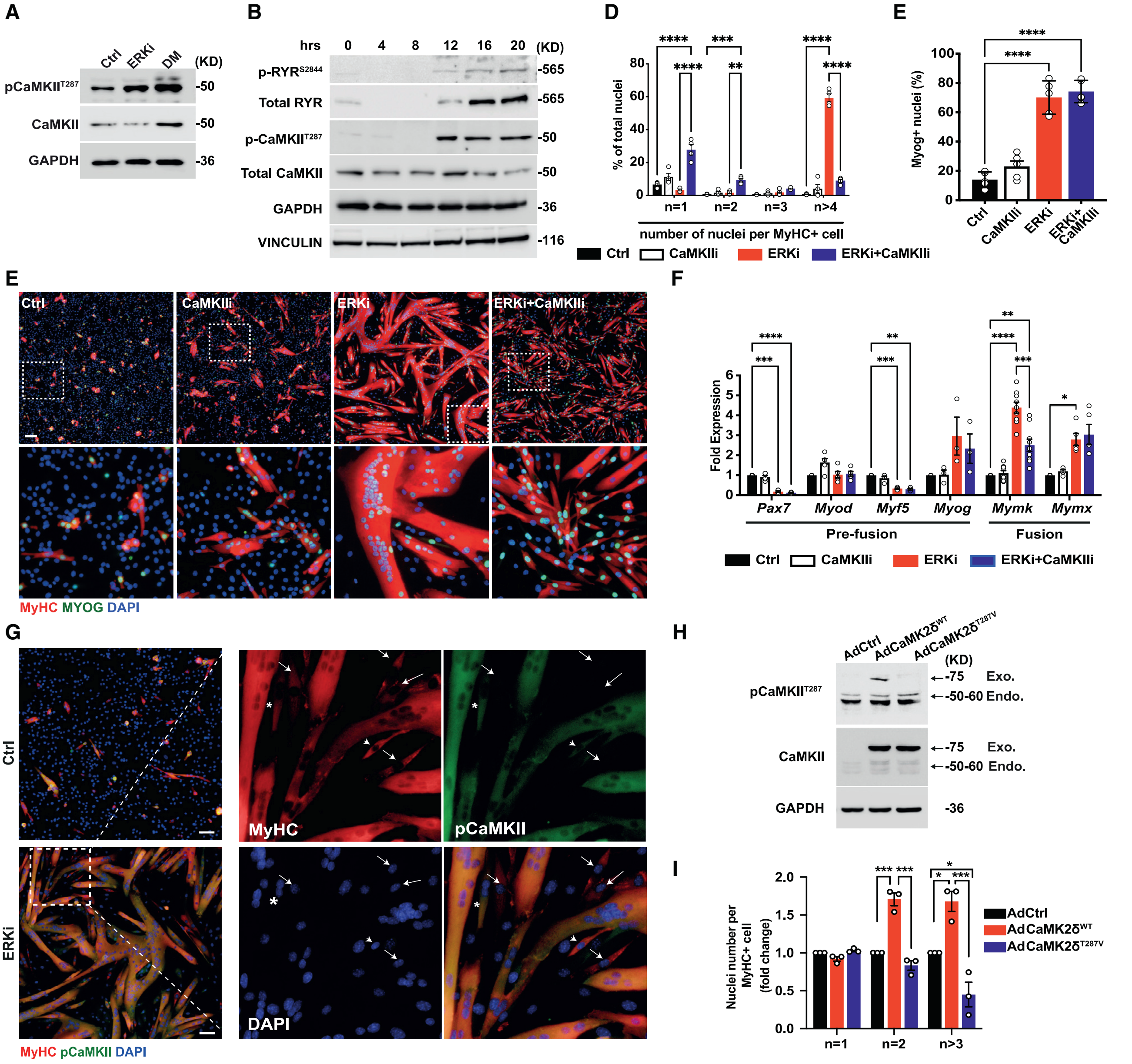

Figure 4. Myotube expansion requires calcium-dependent CaMKII activation

(A) Representative WB of CaMKII activation (T287 phosphorylation) at 24 h after treatment with ERKi or DM.

(B) Representative WB of time-course experiments showing RYR (S2844) and CaMKII (T287) activation following ERKi treatment.

(C) Representative IF images of cells treated with DMSO (Ctrl), 1 μM ERKi, 5 μM KN93 (CaMKIIi), or co-treated with ERKi and CaMKIIi at 24 h. Cells were stained for the differentiation markers MyHC (red), MYOG (green), and DAPI (blue). Indicated regions are enlarged on the bottom.

(D) Fusion index for (C); values are stratified by number of nuclei per MyHC$^+$ fiber. Total number of nuclei assayed n = 61,510.

(E) Quantification of MYOG$^+$ nuclei per field of (C). Total number of nuclei assayed n = 112,901.

(F) qRT-PCR gene-expression analysis of the experiment shown in (E); gene expression was normalized to *Hprt*. Values are expressed as fold change from Ctrl.

(G) Representative IF images showing p-CaMKII localization (green) primarily to myotubes, at 24 h post treatment with ERKi. Indicated region in the ERKi image is enlarged and divided into individual channels on the right. Arrows indicate mononucleated MyHC$^+$ cells, which are negative for p-CaMKII, while the asterisk shows a binucleated MyHC$^+$ cell, which is p-CaMKII$^+$. Arrowhead shows a MyHC$^+$ cell that has already fused with a myotube and is p-CaMKII$^+$.

(H) Representative WB of infection experiments showing activation state of exogenous wild-type CaMKII (Ad-CaMKII^{WT}) or a phospho-null mutant (Ad-CaMKII^{T287V}) expressed in myoblasts 72 h following treatment with DM. Bands for endogenous and exogenous CaMKII are indicated.

(I) Fusion index for the CaMKII infection study at 72 h treatment in DM, presented as fold change from control virus. Total number of nuclei assayed n = 18,758.

Error bars indicate SEM. Scale bars: 100 μm.

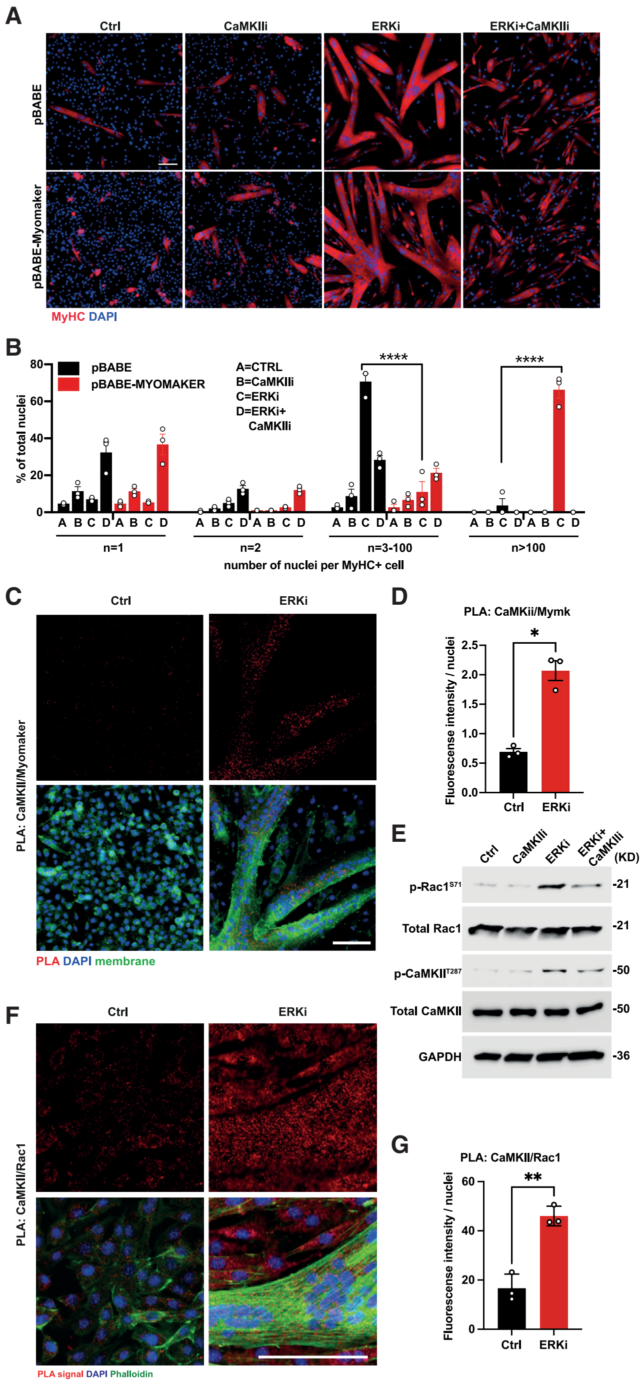

Figure 5. CaMKII interacts with and regulates MYMK and Rac1 during fusion

(A) Representative IF images of myoblasts infected with control retrovirus or virus expressing Myomaker, and treated with DMSO (Ctrl), 1 μM ERKi, 5 μM CaMKIIi, or co-treated with ERKi and CaMKIIi for 18 h.

(B) Stratified fusion index of (A).

(D) Representative images showing proximity ligation assay (PLA) between CaMKII and MYMK for DMSO (Ctrl) or ERKi-treated myoblasts at 24 h post treatment. Top panel shows the PLA signal (red), and bottom panel shows the overlay of PLA signal (red), membrane marker (green), and nuclei (blue).

(E) Quantification of the PLA assay in (D) shown as the mean fluorescent intensity normalized to nuclei number per field. (E) Representative WB analysis of Rac1 S71 phosphorylation following treatment with ERKi and co-treatment with CaMKIIi.

(F) Representative images showing results of the PLA between CaMKII and Rac1 for DMSO (Ctrl) or ERKi-treated myoblasts at 24 h post treatment. Top panel for each shows the PLA signal (red) and the bottom panel shows the overlay of PLA signal (red), phalloidin (green), and nuclei (blue).

(G) Quantification of the results of the PLA assay, shown as the mean fluorescent intensity normalized to nuclei number per field. All data are representative of at least 3 biological repeats. Error bars indicate SEM. Scale bars: 100 μm.

(Figure 6A). By the third day post injury, levels of CaMKII increased in regenerating muscle and remained elevated throughout the 8 days examined; this was accompanied by a peak in CaMKII activation at 5 days post injury (Figure 6A). Following these promising results, we sought to examine the requirement for CaMKII during muscle regeneration. To accomplish this, we generated a tamoxifen-inducible and SC-specific conditional double knockout mouse of the CaMKII δ and γ isoforms (Figures 6B and 6C).

In initial studies, we found that CaMKII protein levels in quiescent SCs are highly stable and not efficiently reduced even 3 months following tamoxifen administration. Moreover, KO in SCs would unlikely alter CaMKII protein levels in the mature muscle fibers. To overcome this obstacle, we implemented a repeat-injury model. We reasoned that the initial round of regeneration would reduce the levels of the highly stable CaMKII protein in the SC pool and ultimately in the regenerated muscle fibers, as the DNA content of the fusing KO myoblasts would be integrated into the fibers. Pax7$^{CreERT/+}$, *CaMK2*$\delta^{fl/fl}/\gamma^{fl/fl}$ (scDKO) or Pax7$^{+/+}$, and *CaMK2*$\delta^{fl/fl}/\gamma^{fl/fl}$ (WT) 4-week-old mice were given tamoxifen to induce Cre/Lox-based gene disruption. When the mice were 12 weeks of age, CTX was administered, and the mice were allowed to fully regenerate for 8 weeks. At 8 weeks post injury, mice were either sacrificed to harvest primary myoblasts from the injured leg (to assess function *in vitro*) or subjected to a second CTX injury and sacrificed 14 days post injury for histological analysis. Reduction in CaMKII levels were indeed validated in scDKO myoblasts harvested 8 weeks following the first injury (Figure 6D). A fusion index demonstrated that such scDKO myoblasts exhibited a significant defect in ERKi-induced secondary fusion compared with those isolated from their WT littermates (Figures 6E and 6F). Specifically, scDKO myoblasts exhibited a loss of the hyperfused myotubes observed in the WT cultures and instead accumulated mononucleated MyHC$^+$ cells and nascent myotubes (Figures 6E and 6F). These results match and recapitulate the observations made on myoblast cultures treated with CaMKIIi. Furthermore, scDKO mice that received repeated injuries had significantly smaller fiber cross-sectional area (851.4 μM^2 ± 37.5) compared with their WT counterparts (975 μM^2 ± 25) (Figures 6G and 6H) and a trend toward more centrally located nuclei (Figure 6I). Taken together, the genetic loss of CaMK2δ/γ is sufficient to impair myoblast fusion and muscle regeneration.

Finally, we tested whether this pathway is conserved beyond mice. To this end, we treated primary chicken myoblasts with ERKi in PM or with the conventional DM for 72 h. By 48 h, fusion was highly elevated in the ERKi-treated cells as compared with DM (fusion index = 64.6% and 8%, respectively) (Figures 6J and 6K). Moreover, ERKi treatment of proliferating chicken

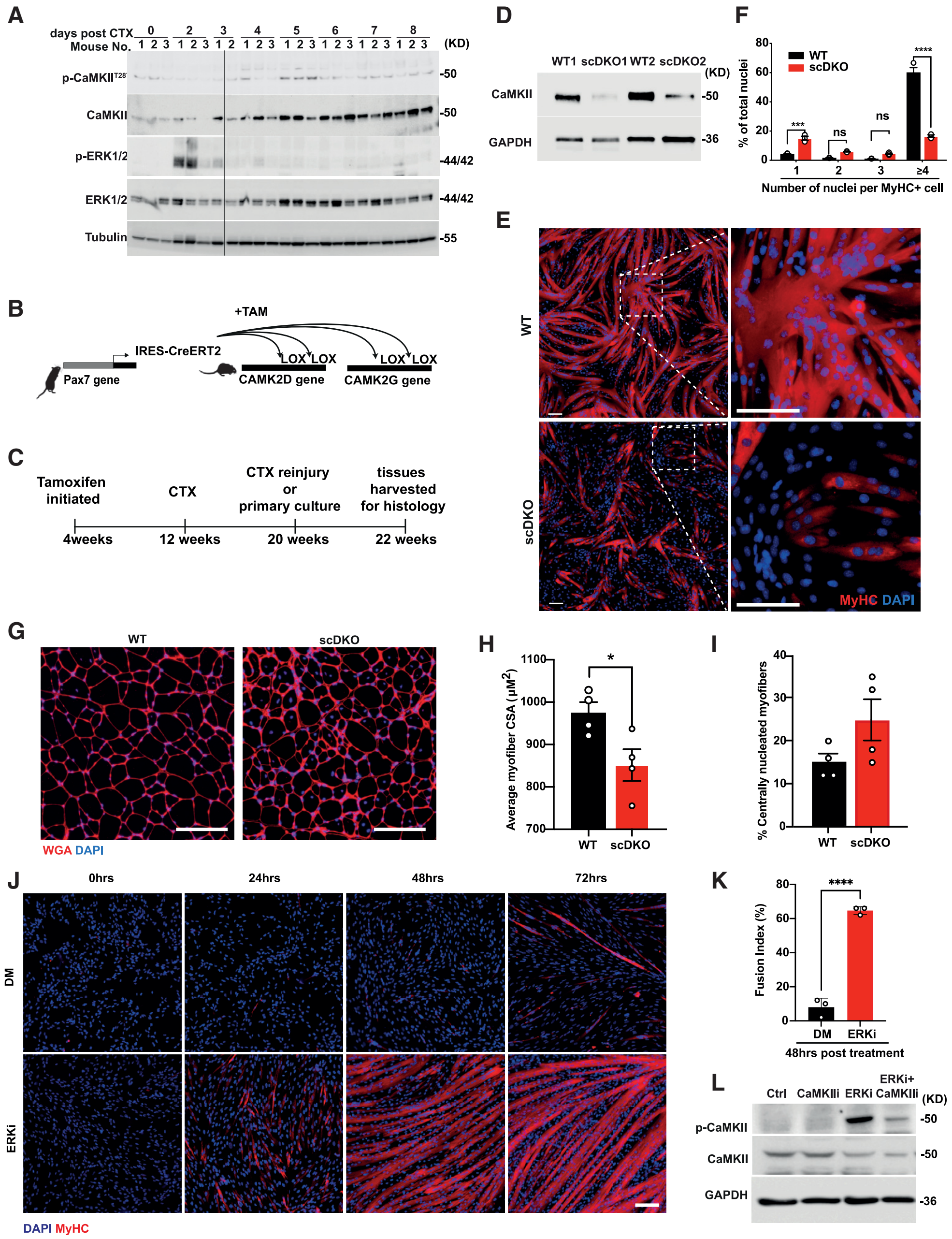

Figure 6. CaMKII function during muscle regeneration and ERK-CaMKII pathway conservation

(A) WB of analysis of indicated proteins from CTX-induced injured muscle. Line indicates where a lane was purposely removed.
(B) Schematic illustration of the SC-specific double CaMKII KO mouse model.
(C) Schematic illustration depicting the timeline of the repeat-injury experimental design.
(D) WB validation of CaMKII depletion in WT or scDKO primary myoblasts isolated for 2 weeks following initial injury.
(E) IF staining of WT or scDKO primary myoblasts following ERKi-induced fusion at 24 h post treatment. Insets are enlarged to the right.
(F) Fusion index comparison between WT (n = 4) and scDKO (n = 4) primary myoblasts stratified by number of nuclei per fiber. Total number of nuclei assayed, n = 12,743.
(G) Representative field of WT and scDKO muscle 14 days after CTX-induced reinjury.
(H) Quantification of myofiber cross-sectional areas of WT (n = 4) and scDKO (n = 4) mice 14 days following reinjury.
(I) Average percentage of central nuclei in WT (n = 4) and scDKO (n = 4) mice 14 days following reinjury. At least 9,000 fibers per mouse were measured for (H) and (I).
(J) Representative IF staining of primary chicken myoblasts over 72 h of treatment either with ERKi in proliferation medium, or in conventional DM.
(K) Fusion index for the 48-h time point of (J).
(L) Representative WB analysis of CaMKII activation in chicken myoblasts, following treatment with ERKi or co-treatment with CaMKIIi. Error bars indicate SEM. All scale bars, 100 μm.

CellPress
OPEN ACCESS

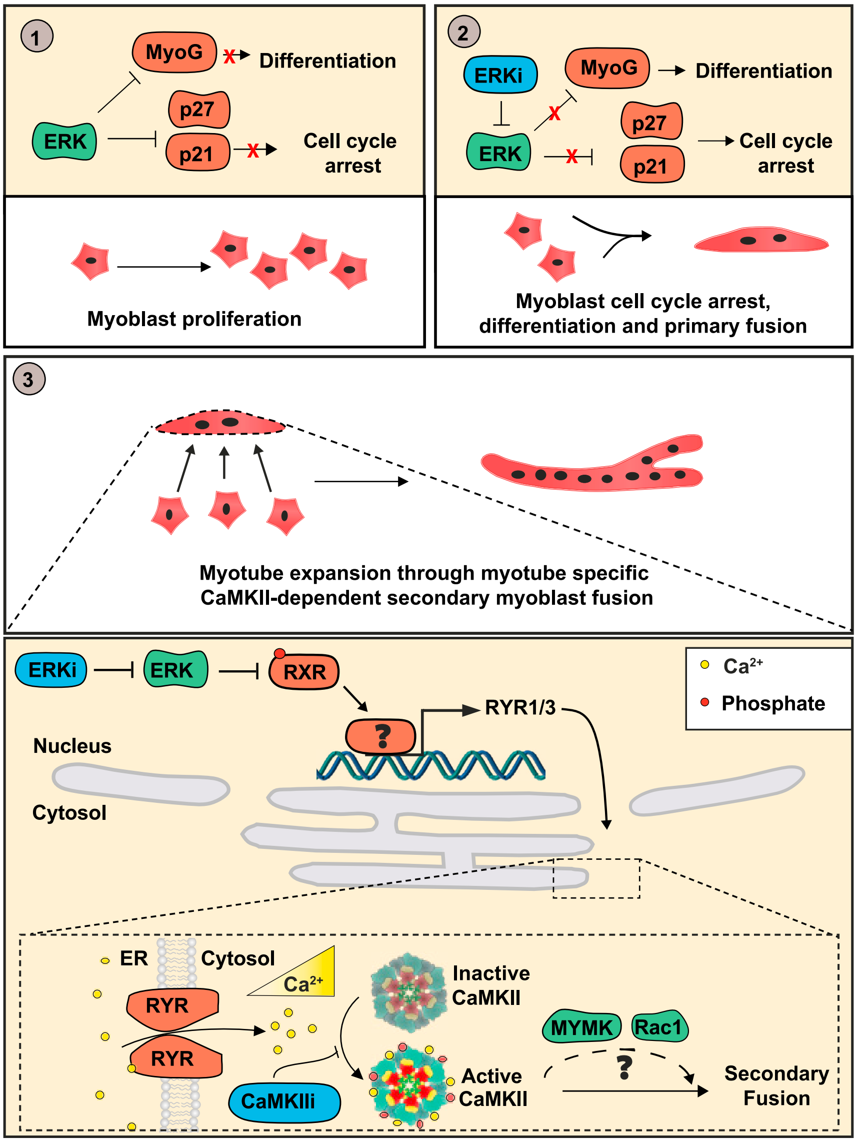

Figure 7. Schematic representation of the ERK1/2-CaMKII secondary fusion pathway

Schematic of the ERK-CaMKII signaling pathway during myoblast differentiation and fusion: (1) In proliferating myoblasts ERK1/2 suppresses MYOG and *p21/p27* activation. (2) Upon ERK1/2 inhibition, *p21/p27* are expressed and cells exit the cell cycle; simultaneously, MYOG is upregulated and cells differentiate. (3) During the differentiation process, ERK1/2 inhibition results in reduced phosphoinhibition of RXR leading to RYR1/3 upregulation and accumulation in early myotubes. RYR activity promotes in Ca^{2+}-dependent CaMKII activation and CaMKII-dependent myotube driven asymmetric fusion, likely through CaMKII regulation of MYMK and Rac1.

myoblasts results in activation of CaMKII (Figure 6L), showing evolutionary conservation in at least two vertebrate lineages.

DISCUSSION

In this study, we demonstrate that ERK1/2 represses processes leading to both differentiation and secondary fusion (Figure 7). We show that ERKi induces robust differentiation and fusion within 24 h, without requiring low serum conditions. ERKi results in reduced RXR phospho-inhibition and in the induction of RXR-dependent RYR expression in nascent myotubes. Whether RXR directly regulates RYR remains to be further explored. Ultimately, RYR accumulation leads to Ca^{2+}-dependent activation of CaMKII in the myotube and to CaMKII-dependent myoblast-to-myotube fusion likely via the interactions with MYMK and Rac1 (Figure 7). In addition, we demonstrate a requirement for CaMKII in muscle regeneration after injury.

The discovery of a signaling cascade that enhances myoblast differentiation and fusion has implications for the ever-growing field of cultivated meat, which builds upon the techniques used for decades of culturing myoblasts (Choi et al., 2021; Post et al., 2020). Here we show that the ERK1/2-CaMKII pathway is conserved in chicken myoblasts, suggesting that it may be conserved in other vertebrates. The cultivated meat industry is actively seeking ways to increase production efficiency in order to reach price-parity with the current meat industry. Therefore, taking advantage of processes that can speed up and enhance efficiency of myoblast differentiation and fusion would facilitate this goal.

The Ca^{2+} channels RYR1 and RYR3 are differentially expressed in skeletal muscle during late development and in different muscle types (Bertocchini et al., 1997; Conti et al., 1996; Tarroni et al., 1997), and mediate fetal myoblast myogenesis *in vitro* (Pisaniello et al., 2003). Here, we demonstrate that elevated expression of *Ryr1* and *Ryr3* during myogenesis are dependent on the activity of ERK and more directly downstream of RXR activity. The delay in the upregulation in RYR protein levels compared with the inhibition of RXR, which occurs within minutes of ERK inhibition, may imply that the regulation of *Ryr* transcription via RXR activity is indirect and that there is yet another intermediate regulator of *Ryr* expression downstream of RXR. As RXR inhibition did not change the number of MYOG positive nuclei, the upregulation of RYR may be dependent on RXR-mediated regulation of MYOD and MYOG function and not expression.

Ca^{2+} has long been implicated in processes regulating myoblast differentiation and fusion (Knudsen and Horwitz, 1977, 1978; Shainberg et al., 1969). Upon ERK inhibition, the activated form of RYR accumulates at the onset of myotube growth. As we show that RYR activity is upstream to CaMKII activation, RYR activation through S2844 phosphorylation is likely regulated by yet another unidentified kinase. Accumulation and phosphorylation of RYR in the cytoplasm of early myotubes likely results in "leakage" of Ca^{2+} from the ER, as previously

reported (Marx et al., 2000; Reiken et al., 2003). Using live imaging of transgenic myoblasts, we observed an acute and persistent increase in the GCaMP6 signal in early myotubes at the onset of secondary fusion and myotube expansion consistent with the activation of CaMKII at the onset of fiber growth.

The fact that ERK1/2 mediates signaling from growth factors and their cognate receptors implies that fusion during muscle development and regeneration is also regulated via long-distance signaling, consistent with recent studies that demonstrated the role of TGF-beta signaling in repressing myoblast fusion (Girardi et al., 2021; Melendez et al., 2021). We show that there is an acute activation of ERK following muscle injury. The direct signal that mediates the activation of ERK in muscle tissue post injury remains unclear. Fibroblast growth factors (FGFs) are potent regulators of myoblast proliferation *in vitro* and *in vivo*, mediated through activation of ERK1/2 (Knight and Kothary, 2011; Pawlikowski et al., 2017). FGF-6 was reported to be elevated in regenerating muscle tissue, and the loss of FGF-6 results in a regeneration defect (Floss et al., 1997), which worsens in FGF-2 and FGF-6 double KO (Neuhaus et al., 2003). Therefore, transient upregulation of FGFs during regeneration may facilitate myoblast proliferation and repression of fusion through ERK activity *in vivo*, and their eventual downregulation may lead to initiation of CaMKII- dependent fusion processes following myoblast cell-cycle exit.

Our study provides direct evidence that fusion in mammalian muscle occurs at a single membrane protrusion extending from an “advancing” myoblast to a “receiving” myotube (Lipton and Konigsberg, 1972; Shilagardi et al., 2013). Live imaging revealed that nascent myotubes (2–3 nuclei) are evident as early as 12 h post treatment with the ERK1/2 inhibitor. RYR upregulation and activation, as well as Ca^{2+}-dependent CaMKII activation also occur after 12 h after treatment with ERKi, concurrent with a concerted increase in myoblast-to-myotube fusion events, leading to rapid growth of the myotube. While the direct role of CaMKII during secondary fusion is not fully understood, its activation precedes fusion and growth of the myotube by a short interval. This temporal link is consistent with the putative interactions of CaMKII with MYMK and Rac1, which are essential for the membrane and cytoskeleton rearrangements needed for fusion (Millay et al., 2013; Vasyutina et al., 2009). Consistently, elevated Rac1 serine 71 phosphorylation following ERK inhibition, a site previously identified for switching the function of Rac1 from being prolamellipodial to a more filopodial-promoting phenotype (Schwarz et al., 2012), is dependent on CaMKII activation. Moreover, MYMK activity in myotubes appears to depend on CaMKII activity. Therefore, one possible role of CaMKII during fusion might be to regulate the preparation of the post synapse on the receiving myotube side through regulation of Myomaker and Rac1.

In summary, we have characterized a pleiotropic role for ERK signaling in muscle biology in the direct and independent repression of cell-cycle exit, differentiation, and secondary fusion and have identified CaMKII as a potent regulator of myoblast fusion with myotubes. These findings and methodological advancements will surely have profound and long-lasting implications for the fields of muscle biology, regenerative medicine, and cultivated meat.

Limitations of the study

While we implicate CaMKII in regeneration *in vivo*, a limitation of our study is that the inducible KO of CaMKIIδ/γ isoforms was performed in SCs rather than in myofibers. To compensate for this, we knocked-down CaMKII in muscle fibers by adopting a double injury model. After the first round of injury, nuclei bearing CaMK2δ/γ KO DNA are incorporated into the regenerated muscle, thus creating a myofiber CaMKII knockdown setting for the next round of injury.

The *in vitro* experiments demonstrating the role of CaMKII during fusion were carried out using a chemical inhibitor of CaMKII. Therefore, we cannot rule out a possible role of CaMKII during primary fusion, as it is possible that the chemical inhibitor did not completely inhibit CaMKII activity. However, this is unlikely given the immunofluorescence data showing the myotube specific localization of RYR and the interaction of CaMKII with MYMK as evident by PLA. Taken together with the observation that CaMKII activation is only evident upon formation of nascent myotubes and not in the mononucleated myocytes, and similarly that KO myoblasts are still able to fuse to form bi- and trinucleated cells *in vitro*, we conclude that the observed effect on regeneration is likely due to an impairment of CaMKII activity in myofibers or *de novo* myotubes *in vivo*, which fail to fuse with the existing myofiber.

STAR★METHODS

Detailed methods are provided in the online version of this paper and include the following:

- KEY RESOURCES TABLE
- RESOURCE AVAILABILITY
  - Lead contact
  - Material availability
  - Data and code availability
- EXPERIMENTAL MODEL AND SUBJECT DETAILS
  - Animal ethics statement
  - *In vivo* experimental animal models
  - Genetic models for primary myoblast cultures and isolation technique
- METHOD DETAILS
  - CTX induced injuries
  - *In vitro* fusion assays of primary myoblast cultures
  - Statistical analysis

SUPPLEMENTAL INFORMATION

Supplemental information can be found online at https://doi.org/10.1016/j.devcel.2021.11.022.

ACKNOWLEDGMENTS

CaMK2$\delta^{fl/fl}$/$\gamma^{fl/fl}$ mice were kindly provided by Eric Olson and Johannes Backs. Histology sections were prepared by Calanit Raanan. This study was supported by grants to E.T. from the AFM (#21655), the Weizmann Institute Hellen and Martin Kimmel Stem Cell grant, the European Research Council (ERC StG #281289, ERC AdG #788194), the Israel Science Foundation (ISF), and Minerva Foundation with funding from the Federal German Ministry for Education and Research (to E.T. and O.A.). This project also received funding from the European Research Council (ERC StG # 851080 to O.A.). O.A. also acknowledges funding from the David Barton Center for Research on the Chemistry of Life and the Ruth and Herman Albert Scholarship Program for New Scientists as well as the Estate of Fannie Sherr. O.A. is an incumbent of the Miriam Berman Presidential Development Chair.

AUTHOR CONTRIBUTIONS

T.E., E.T., and O.A. conceived and designed the experiments. T.E., with help from G.Z., E.A., and S.S., carried out most of the experiments and analyzed the data. Specifically, G.Z. performed and analyzed the live-cell imaging data with assistance from N.S. and S.S. T.E., S.S., and E.A. performed qRT-PCR experiments. T.E., G.Z., and S.S. performed and analyzed the experiments comparing ERKi in PM and DM. T.E. and G.Z. performed immunohistochemistry. Y.Z. and A.Z. performed the simulations. T.E. and K.U. performed CTX injuries, and T.E. carried out all follow-up studies. E.S. contributed to experimental design and critical review of the manuscript. D.M. contributed to the design of the *in vivo* model and associated experiments. E.T. and O.A. supervised the project. T.E, E.T., and O.A. wrote the manuscript with editing contributions from all the authors.

DECLARATION OF INTERESTS

T.E, E.T., and O.A. hold a patent related to the scientific findings presented in this manuscript and are the founders of ProFuse Technology. T.E. is the CTO and E.T. and O.A. are the scientific advisors of ProFuse Technology.

Received: December 8, 2020
Revised: July 28, 2021
Accepted: November 21, 2021
Published: December 20, 2021

REFERENCES

Abmayr, S.M., and Pavlath, G.K. (2012). Myoblast fusion: lessons from flies and mice. Development *139*, 641–656.

Alric, S., Froeschlé, A., Piquemal, D., Carnac, G., and Bonnieu, A. (1998). Functional specificity of the two retinoic acid receptor RAR and RXR families in myogenesis. Oncogene *16*, 273–282.

Alter, J., Rozentzweig, D., and Bengal, E. (2008). Inhibition of myoblast differentiation by tumor necrosis factor α is mediated by c-Jun N-terminal kinase 1 and leukemia inhibitory factor. J. Biol. Chem. *283*, 23224–23234.

Avinoam, O., Fridman, K., Valansi, C., Abutbul, I., Zeev-Ben-Mordehai, T., Maurer, U.E., Sapir, A., Danino, D., Grünewald, K., White, J.M., and Podbilewicz, B. (2011). Conserved eukaryotic fusogens can fuse viral envelopes to cells. Science *332*, 589–592.

Bayer, K.U., Löhler, J., and Harbers, K. (1996). An alternative, nonkinase product of the brain-specifically expressed Ca2+/calmodulin-dependent kinase II alpha isoform gene in skeletal muscle. Mol. Cell. Biol. *16*, 29–36.

Beckett, K., and Baylies, M.K. (2007). 3D analysis of founder cell and fusion competent myoblast arrangements outlines a new model of myoblast fusion. Dev. Biol. *309*, 113–125.

Bennett, A.M., and Tonks, N.K. (1997). Regulation of distinct stages of skeletal muscle differentiation by mitogen-activated protein kinases. Science *278*, 1288–1291.

Bertocchini, F., Ovitt, C.E., Conti, A., Barone, V., Schöler, H.R., Bottinelli, R., Reggiani, C., and Sorrentino, V. (1997). Requirement for the ryanodine receptor type 3 for efficient contraction in neonatal skeletal muscles. EMBO J *16*, 6956–6963.

Campbell, J.S., Wenderoth, M.P., Hauschka, S.D., and Krebs, E.G. (1995). Differential activation of mitogen-activated protein kinase in response to basic fibroblast growth factor in skeletal muscle cells. Proc. Natl. Acad. Sci. USA *92*, 870–874.

Chal, J., and Pourquié, O. (2017). Making muscle: skeletal myogenesis in vivo and in vitro. Development *144*, 2104–2122.

Chen, E.H. (2011). Invasive podosomes and myoblast fusion. Curr. Top. Membr. *68*, 235–258.

Chen, E.H., and Olson, E.N. (2005). Unveiling the mechanisms of cell-cell fusion. Science *308*, 369–373.

Choi, K.-H., Yoon, J.W., Kim, M., Lee, H.J., Jeong, J., Ryu, M., Jo, C., and Lee, C.-K. (2021). Muscle stem cell isolation and in vitro culture for meat production: A methodological review. Compr. Rev. Food Sci. Food Saf. *20*, 429–457.

Constantin, B., Cognard, C., and Raymond, G. (1996). Myoblast fusion requires cytosolic calcium elevation but not activation of voltage-dependent calcium channels. Cell Calcium *19*, 365–374.

Conti, A., Gorza, L., and Sorrentino, V. (1996). Differential distribution of ryanodine receptor type 3 (RyR3) gene product in mammalian skeletal muscles. Biochem. J. *316*, 19–23.

Desgeorges, T., Liot, S., Lyon, S., Bouvière, J., Kemmel, A., Trignol, A., Rousseau, D., Chapuis, B., Gondin, J., Mounier, R., et al. (2019). Open-CSAM, a new tool for semi-automated analysis of myofiber cross-sectional area in regenerating adult skeletal muscle. Skelet. Muscle *9*, 2.

Dinev, D., Jordan, B.W., Neufeld, B., Lee, J.D., Lindemann, D., Rapp, U.R., and Ludwig, S. (2001). Extracellular signal regulated kinase 5 (ERK5) is required for the differentiation of muscle cells. EMBO Rep *2*, 829–834.

Dumont, N.A., and Rudnicki, M.A. (2017). Characterizing satellite cells and myogenic progenitors During skeletal muscle regeneration. Methods Mol. Biol. *1560*, 179–188.

Eilers, W., Gevers, W., van Overbeek, D., de Haan, A., Jaspers, R.T., Hilbers, P.A., van Riel, N., and Flück, M. (2014a). CaMKII content affects contractile, but not mitochondrial, characteristics in regenerating skeletal muscle. BMC Physiol *14*, 1215.

Eilers, W., Jaspers, R.T., de Haan, A., Ferrié, C., Valdivieso, P., and Flück, M. (2014b). Muscle-type specific autophosphorylation of CaMKII isoforms after paced contractions. Biomed Res Int *2014*, 1–20.

Floss, T., Arnold, H.H., and Braun, T. (1997). A role for FGF-6 in skeletal muscle regeneration. Genes Dev *11*, 2040–2051.

Froeschlé, A., Alric, S., Kitzmann, M., Carnac, G., Auradé, F., Rochette-Egly, C., and Bonnieu, A. (1998). Retinoic acid receptors and muscle b-HLH proteins: partners in retinoid-induced myogenesis. Oncogene *16*, 3369–3378.

Girardi, F., Taleb, A., Ebrahimi, M., Datye, A., Gamage, D.G., Peccate, C., Giordani, L., Millay, D.P., Gilbert, P.M., Cadot, B., and Le Grand, F. (2021). TGFβ signaling curbs cell fusion and muscle regeneration. Nat. Commun. *12*, 750.

Harel, I., Nathan, E., Tirosh-Finkel, L., Zigdon, H., Guimarães-Camboa, N., Evans, S.M., and Tzahor, E. (2009). Distinct origins and genetic programs of head muscle satellite cells. Dev. Cell *16*, 822–832.

Hernández, J.M., and Podbilewicz, B. (2017). The hallmarks of cell-cell fusion. Development *144*, 4481–4495.

Hernández-Hernández, J.M., García-González, E.G., Brun, C.E., and Rudnicki, M.A. (2017). The myogenic regulatory factors, determinants of muscle development, cell identity and regeneration. Semin. Cell Dev. Biol. *72*, 10–18.

Hindi, S.M., Tajrishi, M.M., and Kumar, A. (2013). Signaling mechanisms in mammalian myoblast fusion. Sci. Signal. *6*, re2.

Horsley, V., Jansen, K.M., Mills, S.T., and Pavlath, G.K. (2003). IL-4 acts as a myoblast recruitment factor during mammalian muscle growth. Cell *113*, 483–494.

Jones, N.C., Fedorov, Y.V., Rosenthal, R.S., and Olwin, B.B. (2001). ERK1/2 is required for myoblast proliferation but is dispensable for muscle gene expression and cell fusion. J. Cell. Physiol. *186*, 104–115.

Khilji, S., Hamed, M., Chen, J., and Li, Q. (2020). Dissecting myogenin-mediated retinoid X receptor signaling in myogenic differentiation. Commun. Biol. *3*, 315.

Kim, J.H., and Chen, E.H. (2019). The fusogenic synapse at a glance. J. Cell Sci. *132*, jcs213124.

Kim, J.H., Ren, Y., Ng, W.P., Li, S., Son, S., Kee, Y.S., Zhang, S., Zhang, G., Fletcher, D.A., Robinson, D.N., and Chen, E.H. (2015). Mechanical tension drives cell membrane fusion. Dev. Cell *32*, 561–573.

Knight, J.D.R., and Kothary, R. (2011). The myogenic kinome: protein kinases critical to mammalian skeletal myogenesis. Skelet. Muscle *1*, 29.

Knudsen, K.A., and Horwitz, A.F. (1977). Tandem events in myoblast fusion. Dev. Biol. *58*, 328–338.

Knudsen, K.A., and Horwitz, A.F. (1978). Differential inhibition of myoblast fusion. Dev. Biol. *66*, 294–307.

Kreusser, M.M., Lehmann, L.H., Keranov, S., Hoting, M.-O., Oehl, U., Kohlhaas, M., Reil, J.-C., Neumann, K., Schneider, M.D., Hill, J.A., et al. (2014). Cardiac CaM kinase II genes δ and γ contribute to adverse remodeling but redundantly inhibit calcineurin-induced myocardial hypertrophy. Circulation *130*, 1262–1273.

Le May, M., Mach, H., Lacroix, N., Hou, C., Chen, J., and Li, Q. (2011). Contribution of retinoid X receptor signaling to the specification of skeletal muscle lineage. J. Biol. Chem. *286*, 26806–26812.

Lee, D.M., and Chen, E.H. (2019). Drosophila myoblast fusion: invasion and resistance for the ultimate union. Annu. Rev. Genet. *53*, 67–91.

Lehka, L., and Rędowicz, M.J. (2020). Mechanisms regulating myoblast fusion: a multilevel interplay. Semin. Cell Dev. Biol. *104*, 81–92.

Leikina, E., Gamage, D.G., Prasad, V., Goykhberg, J., Crowe, M., Diao, J., Kozlov, M.M., Chernomordik, L.V., and Millay, D.P. (2018). Myomaker and Myomerger work independently to control distinct steps of membrane remodeling during myoblast fusion. Dev. Cell *46*, 767–780.e7.

Lipton, B.H., and Konigsberg, I.R. (1972). A fine-structural analysis of the fusion of myogenic cells. J. Cell Biol. *53*, 348–364.

Luo, J., Deng, Z.-L., Luo, X., Tang, N., Song, W.-X., Chen, J., Sharff, K.A., Luu, H.H., Haydon, R.C., Kinzler, K.W., et al. (2007). A protocol for rapid generation of recombinant adenoviruses using the AdEasy system. Nat. Protoc. *2*, 1236–1247.

Macoritto, M., Nguyen-Yamamoto, L., Huang, D.C., Samuel, S., Yang, X.F., Wang, T.T., White, J.H., and Kremer, R. (2008). Phosphorylation of the human retinoid X receptor alpha at serine 260 impairs coactivator(s) recruitment and induces hormone resistance to multiple ligands. J. Biol. Chem. *283*, 4943–4956.

Marx, S.O., Reiken, S., Hisamatsu, Y., and Jayaraman, T. (2000). PKA phosphorylation dissociates FKBP12. 6 from the calcium release channel (ryanodine receptor): defective regulation in failing hearts. Cell *101*, 365–376.

Matsushima-Nishiwaki, R., Okuno, M., Adachi, S., Sano, T., Akita, K., Moriwaki, H., Friedman, S.L., and Kojima, S. (2001). Phosphorylation of retinoid X receptor alpha at serine 260 impairs its metabolism and function in human hepatocellular carcinoma. Cancer Res. *61*, 7675–7682.

Melendez, J., Sieiro, D., Salgado, D., Morin, V., Dejardin, M.-J., Zhou, C., Mullen, A.C., and Marcelle, C. (2021). TGFβ signalling acts as a molecular brake of myoblast fusion. Nat. Commun. *12*, 749.

Michailovici, I., Harrington, H.A., Azogui, H.H., Yahalom-Ronen, Y., Plotnikov, A., Ching, S., Stumpf, M.P.H., Klein, O.D., Seger, R., and Tzahor, E. (2014). Nuclear to cytoplasmic shuttling of ERK promotes differentiation of muscle stem/progenitor cells. Development *141*, 2611–2620.

Millay, D.P., Gamage, D.G., Quinn, M.E., Min, Y.-L., Mitani, Y., Bassel-Duby, R., and Olson, E.N. (2016). Structure-function analysis of myomaker domains required for myoblast fusion. Proc. Natl. Acad. Sci. USA *113*, 2116–2121.

Millay, D.P., O'Rourke, J.R., Sutherland, L.B., Bezprozvannaya, S., Shelton, J.M., Bassel-Duby, R., and Olson, E.N. (2013). Myomaker is a membrane activator of myoblast fusion and muscle formation. Nature *499*, 301–305.

Millay, D.P., Sutherland, L.B., Bassel-Duby, R., and Olson, E.N. (2014). Myomaker is essential for muscle regeneration. Genes Dev. *28*, 1641–1646.

Moradi, F., Copeland, E.N., Baranowski, R.W., Scholey, A.E., Stuart, J.A., and Fajardo V, A. (2020). Calmodulin-binding proteins in muscle: a minireview on nuclear receptor interacting protein, neurogranin, and growth-associated protein 43. Int J Mol Sci. *21*, 1016.

Morris, E.J., Jha, S., Restaino, C.R., Dayananth, P., Zhu, H., Cooper, A., Carr, D., Deng, Y., Jin, W., Black, S., et al. (2013). Discovery of a novel ERK inhibitor with activity in models of acquired resistance to BRAF and MEK inhibitors. Cancer Discov. *3*, 742–750.

Murphy, M.M., Lawson, J.A., Mathew, S.J., Hutcheson, D.A., and Kardon, G. (2011). Satellite cells, connective tissue fibroblasts and their interactions are crucial for muscle regeneration. Development *138*, 3625–3637.

Nakanishi, K., Kakiguchi, K., Yonemura, S., Nakano, A., and Morishima, N. (2015). Transient Ca2+ depletion from the endoplasmic reticulum is critical for skeletal myoblast differentiation. FASEB J. *29*, 2137–2149.

Neuhaus, P., Oustanina, S., Loch, T., Krüger, M., Bober, E., Dono, R., Zeller, R., and Braun, T. (2003). Reduced mobility of fibroblast growth factor (FGF)-deficient myoblasts might contribute to dystrophic changes in the musculature of FGF2/FGF6/mdx triple-mutant mice. Mol. Cell. Biol. *23*, 6037–6048.

Nissan, M.H., Rosen, N., and Solit, D.B. (2013). ERK pathway inhibitors: how low should we go? Cancer Discov. *3*, 719–721.

Ojuka, E.O., Goyaram, V., and Smith J, A. (2012). The role of CaMKII in regulating GLUT4 expression in skeletal muscle. Am J Physiol Endocrinol Metab *303*, E3221–E331.

Önel, S.-F., and Renkawitz-Pohl, R. (2009). FuRMAS: triggering myoblast fusion in Drosophila. Dev. Dyn. *238*, 1513–1525.

Pawlikowski, B., Vogler, T.O., Gadek, K., and Olwin, B.B. (2017). Regulation of skeletal muscle stem cells by fibroblast growth factors. Dev. Dyn. *246*, 359–367.

Pisaniello, A., Serra, C., Rossi, D., Vivarelli, E., Sorrentino, V., Molinaro, M., and Bouché, M. (2003). The block of ryanodine receptors selectively inhibits fetal myoblast differentiation. J. Cell Sci. *116*, 1589–1597.

Post, M.J., Levenberg, S., Kaplan, D.L., Genovese, N., Fu, J., Bryant, C.J., Negowetti, N., Verzijden, K., and Moutsatsou, P. (2020). Scientific, sustainability and regulatory challenges of cultured meat. Nat. Food *1*, 403–415.

Quinn, M.E., Goh, Q., Kurosaka, M., Gamage, D.G., Petrany, M.J., Prasad, V., and Millay, D.P. (2017). Myomerger induces fusion of non-fusogenic cells and is required for skeletal muscle development. Nat. Commun. *8*, 15665.

Rau, A., Buttgereit, D., Holz, A., Fetter, R., Doberstein, S.K., Paululat, A., Staudt, N., Skeath, J., Michelson, A.M., and Renkawitz-Pohl, R. (2001). rolling pebbles (rols) is required in Drosophila muscle precursors for recruitment of myoblasts for fusion. Development *128*, 5061–5073.

Reiken, S., Lacampagne, A., Zhou, H., Kherani, A., Lehnart, S.E., Ward, C., Huang, F., Gaburjakova, M., Gaburjakova, J., Rosemblit, N., et al. (2003). PKA phosphorylation activates the calcium release channel (ryanodine receptor) in skeletal muscle: defective regulation in heart failure. J. Cell Biol. *160*, 919–928.

Richter, E.A., and Hargreaves, M. (2013). Exercise, GLUT4, and skeletal muscle glucose uptake. Physiol Rev. *93*, 999–1017.

Riedl, J., Crevenna, A.H., Kessenbrock, K., Yu, J.H., Neukirchen, D., Bista, M., Bradke, F., Jenne, D., Holak, T.A., Werb, Z., et al. (2008). Lifeact: a versatile marker to visualize F-actin. Nat. Methods *5*, 605–607.

Rochlin, K., Yu, S., Roy, S., and Baylies, M.K. (2010). Myoblast fusion: when it takes more to make one. Dev. Biol. *341*, 66–83.

Rose, A.J., Frøsig, C., Kiens, B., Wojtaszewski, J.F., and Richter E, A. (2007). Effect of endurance exercise training on Ca2+–calmodulin-dependent protein kinase II expression and signalling in skeletal muscle of humans. J Physiol *583*, 785–795.

Sarbassov, D.D., Jones, L.G., and Peterson, C.A. (1997). Extracellular signal-regulated kinase-1 and -2 respond differently to mitogenic and differentiative signaling pathways in myoblasts. Mol. Endocrinol. *11*, 2038–2047.

Sarbassov, D.D., and Peterson, C.A. (1998). Insulin receptor substrate-1 and phosphatidylinositol 3-kinase regulate extracellular signal-regulated kinase-dependent and -independent signaling pathways during myogenic differentiation. Mol. Endocrinol. *12*, 1870–1878.

Scata, K.A., Bernard, D.W., Fox, J., and Swain, J.L. (1999). FGF receptor availability regulates skeletal myogenesis. Exp. Cell Res. *250*, 10–21.

Schejter, E.D. (2016). Myoblast fusion: experimental systems and cellular mechanisms. Semin. Cell Dev. Biol. *60*, 112–120.

Schmidt, M., Schüler, S.C., Hüttner, S.S., von Eyss, B., and von Maltzahn, J. (2019). Adult stem cells at work: regenerating skeletal muscle. Cell. Mol. Life Sci. *76*, 2559–2570.

Schneider, C.A., Rasband, W.S., and Eliceiri, K.W. (2012). NIH image to ImageJ: 25 years of image analysis. Nat. Methods *9*, 671–675.

Schwarz, J., Proff, J., Hävemeier, A., Ladwein, M., Rottner, K., Barlag, B., Pich, A., Tatge, H., Just, I., and Gerhard, R. (2012). Serine-71 phosphorylation of Rac1 modulates downstream signaling. PLoS One *7*, e44358.

CellPress
OPEN ACCESS

Segal, D., Dhanyasi, N., Schejter, E.D., and Shilo, B.-Z. (2016). Adhesion and fusion of muscle cells are promoted by filopodia. Dev. Cell *38*, 291–304.

Segalés, J., Perdiguero, E., and Muñoz-Cánoves, P. (2016). Regulation of muscle stem cell functions: a focus on the p38 MAPK signaling pathway. Front. Cell Dev. Biol. *4*, 91.

Shainberg, A., Yagil, G., and Yaffe, D. (1969). Control of myogenesis in vitro by Ca2+ concentration in nutrittonal medium. Exp. Cell Res. *58*, 163–167.

Shi, C., Cai, Y., Li, Y., Li, Y., Hu, N., Ma, S., Hu, S., Zhu, P., Wang, W., and Zhou, H. (2018). Yap promotes hepatocellular carcinoma metastasis and mobilization via governing cofilin/F-actin/lamellipodium axis by regulation of JNK/Bnip3/SERCA/CaMKII pathways. Redox Biol. *14*, 59–71.

Shilagardi, K., Li, S., Luo, F., Marikar, F., Duan, R., Jin, P., Kim, J.H., Murnen, K., and Chen, E.H. (2013). Actin-propelled invasive membrane protrusions promote fusogenic protein engagement during cell-cell fusion. Science *340*, 359–363.

Stringer, C., Wang, T., Michaelos, M., and Pachitariu, M. (2021). Cellpose: a generalist algorithm for cellular segmentation. Nat. Methods *18*, 100–106.

Sunadome, K., Yamamoto, T., Ebisuya, M., Kondoh, K., Sehara-Fujisawa, A., and Nishida, E. (2011). ERK5 regulates muscle cell fusion through Klf transcription factors. Dev. Cell *20*, 192–205.

Tarroni, P., Rossi, D., Conti, A., and Sorrentino, V. (1997). Expression of the ryanodine receptor type 3 calcium release channel during development and differentiation of mammalian skeletal muscle cells. J. Biol. Chem. *272*, 19808–19813.

Tiffin, N., Adi, S., Stokoe, D., Wu, N.-Y., and Rosenthal, S.M. (2004). Akt phosphorylation is not sufficient for insulin-like growth factor-stimulated myogenin expression but must be accompanied by down-regulation of mitogen-activated protein kinase/extracellular signal-regulated kinase phosphorylation. Endocrinology *145*, 4991–4996.

Vasyutina, E., Martarelli, B., Brakebusch, C., Wende, H., and Birchmeier, C. (2009). The small G-proteins Rac1 and Cdc42 are essential for myoblast fusion in the mouse. Proc. Natl. Acad. Sci. USA *106*, 8935–8940.

Vest, R.S., Davies, K.D., O'Leary, H., Port, J.D., and Bayer, K.U. (2007). Dual mechanism of a natural CaMKII inhibitor. Mol. Biol. Cell *18*, 5024–5033.

Wang, C., Xu, H., Lin, S., Deng, W., Zhou, J., Zhang, Y., Shi, Y., Peng, D., and Xue, Y. (2020). GPS 5.0: an update on the prediction of kinase-specific phosphorylation sites in proteins. Genomics Proteomics Bioinformatics *18*, 72–80.

Wu, Z., Woodring, P.J., Bhakta, K.S., Tamura, K., Wen, F., Feramisco, J.R., Karin, M., Wang, J.Y., and Puri, P.L. (2000). p38 and extracellular signal-regulated kinases regulate the myogenic program at multiple steps. Mol. Cell. Biol. *20*, 3951–3964.

Xie, S.-J., Li, J.-H., Chen, H.-F., Tan, Y.-Y., Liu, S.-R., Zhang, Y., Xu, H., Yang, J.-H., Liu, S., Zheng, L.-L., et al. (2018). Inhibition of the JNK/MAPK signaling pathway by myogenesis-associated miRNAs is required for skeletal muscle development. Cell Death Differ. *25*, 1581–1597.

Yang, W., Chen, Y., Zhang, Y., Wang, X., Yang, N., and Zhu, D. (2006). Extracellular signal-regulated kinase 1/2 mitogen-activated protein kinase pathway is involved in myostatin-regulated differentiation repression. Cancer Res. *66*, 1320–1326.

Yohe, M.E., Gryder, B.E., Shern, J.F., Song, Y.K., Chou, H.C., Sindiri, S., Mendoza, A., Patidar, R., Zhang, X., Guha, R., et al. (2018). MEK inhibition induces MYOG and remodels super-enhancers in RAS-driven rhabdomyosarcoma. Sci Transl Med *10*, 828–851.

Zhu, G.-H., Huang, J., Bi, Y., Su, Y., Tang, Y., He, B.-C., He, Y., Luo, J., Wang, Y., Chen, L., et al. (2009). Activation of RXR and RAR signaling promotes myogenic differentiation of myoblastic C2C12 cells. Differentiation *78*, 195–204.

STAR★METHODS

KEY RESOURCES TABLE

REAGENT or RESOURCE	SOURCE	IDENTIFIER
**Antibodies**		
Mouse monoclonal anti-MyHC (MF-20)	DSHB	Cat# MF 20; RRID:AB_2147781
Mouse monoclonal anti-MyHC (MY-32)	Abcam	Cat# ab51263; RRID:AB_2297993
Mouse monoclonal anti-MYOG	Santa Cruz Biotechnology	Cat# sc-13137; RRID:AB_627979
Rabbit polyclonal anti-PH3	Abcam	Cat# ab4729; RRID:AB_880448
Rabbit monoclonal anti-Ki-67	Cell Marque	Cat#275R; RRID:AB_1158033
Mouse monoclonal anti-RYR	Abcam	Cat# ab2868; RRID:AB_2183051
Rabbit polyclonal anti-p-RYR	Abcam	Cat# ab59225; RRID:AB_946327
Rabbit polyclonal anti-p-CAMKII	MERCK	Cat# SAB4504356
Rabbit polyclonal anti-p-CAMKII	Abcam	Cat# ab182647
Rabbit monoclonal anti-CaMKII	Abcam	Cat# ab52476; RRID:AB_868641
Rabbit polyclonal anti-CaMKII	Cell Signaling	Cat# 3362; RRID:AB_2067938
Mouse monoclonal anti-CaMKII	Santa Cruz Biotechnology	Cat# sc-5306; RRID:AB_626788
Rabbit polyclonal anti-ERK1/2	MERCK	Cat# M7927; RRID:AB_260665
Rabbit polyclonal anti-ERK1/2	MERCK	Cat# M5670; RRID:AB_477216
Mouse monoclonal anti-p-ERK1/2	MERCK	Cat# M9692; RRID:AB_260729
Mouse monoclonal anti-Rac1	Millipore	Cat# 05-389; RRID:AB_309712)
Rabbit polyclonal anti-p-Rac1	Millipore	Cat# 07-896-I; RRID:AB_612043
Mouse monoclonal anti-Vinculin	Benny Geiger, Weizmann Institute of Science	
Rabbit monoclonal anti-GAPDH	Abcam	Cat# ab181602; RRID:AB_2630358
Rabbit polyclonal anti-RXRa	Santa Cruz Biotechnology	Cat# sc-553; RRID:AB_2184874
Rabbit polyclonal anti- p-RXR	Affinity Biosciences	Cat# AF8214; RRID:AB_2840276
Rabbit polyclonal anti-TMEM8C	MERCK	Cat# HPA051846 RRID:AB_2681636
**Chemicals, peptides, and recombinant proteins**		
SCH772984	Cayman Chemicals	Cat# 19166
HX-531	Cayman Chemicals	Cat# 20762
Dantrolene	Cayman Chemicals	Cat# 14326
KN93	Cayman Chemicals	Cat# 13319
Tat-scramble (Myr-YGRKKRRQRRRLSGPIIPRRD GRKQRKEDVVK	Peptide 2.0	
Tat-CN21 (Myr-YGRKKRRQRRRKRPPKL GQIGRSKRVVIEDDR	Peptide 2.0	
tamoxifen	SIGMA	Cat# T5648
Cardiotoxin (CTX)	Lotaxan	Cat# L8102
**Critical commercial assays**		
Duolink Proximity Ligation Assay	MERCK	Cat# DUO92013<br>Cat# DUO92005; RRID:AB_2810942<br>Cat# DUO92001; RRID:AB_2810939
**Deposited data**		
DOI: https://zenodo.org/badge/latestdoi/284677675		
**Experimental models: Organisms/strains**		
Mouse: B6.Cg-*Pax7*$^{tm1(cre/ERT2)Gaka}$/J	The Jackson laboratory	017763
Mouse: CaMK2D^{flox}/G^{flox}	Eric Olson/Johannes Backs	

(*Continued on next page*)

Continued

REAGENT or RESOURCE	SOURCE	IDENTIFIER
Mouse: B6J.Cg-*Gt(ROSA)26Sor*$^{tm96(CAG-GCaMP6s)Hze}$/MwarJ	The Jackson laboratory	028866
Mouse: ROSA26-tdTomato	Weizmann Institute mouse repository	
Mouse: nuclear reporter nTnG - B6N.129S6-*Gt(ROSA)26Sor*$^{tm1(CAG-tdTomato*,-EGFP*)Ees}$/J	The Jackson laboratory	023537
Mouse: membrane reporter mTmG - STOCK *Gt(ROSA)26Sor*$^{tm4(ACTB-tdTomato,-EGFP)Luo}$/J	The Jackson laboratory	007576
Mouse: LifeActGFP	Weizmann Institute mouse repository	
Mouse: c57/bl6OlaHsd	Envigo	
Oligonucleotides		
Primers for qRT-PCR (see Table S1)	This paper	
Primers for cloning (see Table S2)	This paper	
Recombinant DNA		
RedTrack-CMV-EGFP-FLAG-CAMK2D^{WT} (Ad-CaMK2D^{WT})	This paper	
RedTrack-CMV-EGFP-FLAG-CAMK2D^{T287V} (Ad-CaMK2D^{T287V})	This paper	
RedTrack-CMV (Ad-Ctrl)	Addgene	Cat# 50957
pBabe-MYMK-CFPnls	This paper	
pBabe-CFPnls	This paper	
pBabe-dsRed plasmid	This paper	
Software and algorithms		
Open-CSAM, semi-automated analysis tool with ImageJ		
ImageJ v1.52 software	NIH	RRID:SCR_003070
Image Lab software	Bio-Rad	RRID:SCR_014210
StepOne software	Applied Biosystems	RRID:SCR_014281
NIS-Elements imaging software ver.5.11.00	Nikon	RRID:SCR_014329
VisiView software	Visitron Systems GmbH	
Cellpose software		RRID:SCR_021716

RESOURCE AVAILABILITY

Lead contact

Request for reagents and data should be directed to and will be fulfilled by the lead contact, Eldad Tzahor (eldad.tzahor@weizmann.ac.il)

Material availability

All plasmids generated in this study are available upon request to the lead contact.

Data and code availability

- All data reported in this paper will be shared by the lead contact upon request.
- All original code and related data has been deposited at https://github.com/assafZaritskyLab/MyocytesFusionSimulationsGeneratorAnalyzer#readme and is publicly available as of the date of publication. DOIs are listed in the key resources table.
- Any additional information required to reanalyze the data reported in this paper is available from the lead contact upon request.

EXPERIMENTAL MODEL AND SUBJECT DETAILS

Animal ethics statement

All experiments were approved by the Animal Care and Use Committee of the Weizmann Institute of Science (IACUC application # 00720120-4 and 13780519-1). The study is compliant with all of the relevant ethical regulations regarding animal research. The mice

were given ad libitum access to water and food and monitored daily for health and activity. Mice belonging to different experimental groups were caged together in 12-h light/dark cycles and treated the same.

In vivo experimental animal models

To generate satellite cell specific and tamoxifen inducible CaMK2δ/γ double KO mice were, Pax7-Cre^{ERT} mice (Murphy et al., 2011) The Jackson laboratory, stock no. 017763) with double floxed *CaMK2δ$^{fl/fl}$/γ$^{fl/fl}$ mice* (Kreusser et al., 2014). Female Pax7$^{CreERT/+}$; *CaMK2δ$^{fl/fl}$/γ$^{fl/fl}$* (scDKO) or Pax7$^{+/+}$; *CaMK2δ$^{fl/fl}$/γ$^{fl/fl}$* (WT) littermates received intraperitoneal tamoxifen administration beginning at weening (4 weeks of age) for 6 consecutive days, followed by weekly boosters until 12 weeks of age. Then these mice underwent CTX induced injuries described below. 7-week-old Female Wildtype c57/bl6 mice were purchased from ENVIGO, and used to evaluate ERK and CaMKII protein levels following CTX-induced injuries.

Genetic models for primary myoblast cultures and isolation technique

Nuclear and membrane reporter mice were bred inhouse by crossing nTnG$^{+/+}$ and mTmG$^{+/+}$ mice (The Jackson laboratory, stock no 023537, 007576 respectively). Actin/nuclear reporter mice were bred inhouse by crossing LifeAct-GFP mice (Riedl et al., 2008) with nTnG$^{+/+}$ mice. Validation of of transgene expression was performed through examination of ear notch samples under a fluorescence microscope. Ca^{2+} reporter mice were bred inhouse by crossing *Pax7-Cre$^{ERT+/+}$* (The Jackson laboratory, stock no. 017763) with GCaMP6s$^{flstop/flstop}$ *mice* (The Jackson laboratory, stock no. and 028866), tdTomato reporter mice were bred inhouse by crossing *Pax7-Cre$^{ERT+/+}$* with tdTomato$^{flstop/flstop}$. Genotyping was performed on every litter.

Primary mouse myoblasts were isolated from gastrocnemius muscle of female mice, or primary chicken myoblasts were isolated from breast and leg muscles of post-mortem P1 chick breast and leg muscles. Briefly, muscle tissues were incubated in Trypsin B (Biological Industries, Israel) and subjected to mechanical dissociation with a serological pipet. Supernatants were strained and centrifuged. Pellets were resuspended in proliferation media and plated on 10% Matrigel-coated plates at 37° and 5% CO_2 (Harel et al., 2009). For all *in vitro* experiments, proliferation medium was Bio-Amf2 (Biological Industries, Israel) and Differentiation medium (DM) was DMEM:F12 supplemented with 2% horse serum with 1% pen/strep mix. Myoblasts were maintained in proliferation media until reaching approximately 80% confluency and then detached with Trypsin C (Biological Industries, Israel) and subjected to two rounds of pre-plating on uncoated plates to reduce the number of fibroblasts, then seeded for specific experiments. Myoblast isolations from *Pax7-Cre$^{ERT+/+}$*;GCaMP6s$^{flstop/flstop}$ *mice*, and *Pax7-Cre$^{ERT+/+}$*;tdTomato$^{flstop/flstop}$ mice were treated with 5uM of Tamoxifen in culture for 24 hours immediately following harvesting, and fresh proliferation media with 5uM tamoxifen was replaced after 24 hours. Then fresh media was replaced daily without tamoxifen. All *in vitro* experiments with primary myoblasts were done on cells limited to the first and second passage.

METHOD DETAILS

CTX induced injuries

Mice were anesthetized with isoflurane and injected in the right gastrocnemius muscle with CTX dissolved in PBS at 10 sites (3ul per site) at 10μm, using a Hamilton syringe. All injuries were performed on female mice. For mice that received a repeat injury: following the first injury, mice were maintained for an additional 8 weeks and then injured again in the right gastrocnemius, as described above.

In vitro fusion assays of primary myoblast cultures

Primary myoblasts were plated at a density of 8x10^3 per well in 10% Matrigel-coated 96-well plates in proliferation medium for 24 hours. The following day, proliferation media was replaced either with proliferation media containing DMSO (Ctrl) or 1μM ERK1/2 inhibitor (ERKi; SCH772984, Cayman Chemicals), 20μM RXR antagonist (RXRi; HX-531, Cayman Chemicals), 50μM Ryanodine receptor antagonist (RYRi; Dantrolene, Cayman Chemicals), 5μM CaMKII inhibitor (CaMKIIi; KN93, Cayman Chemicals), 50 μM Tat-scramble, and 50 μM Tat- ((Vest et al., 2007) peptide 2.0), or with DM. Inhibitors were used at a the highest concentration before becoming toxic, as determined by a dose response experiments.

Immunofluorescence staining

First passage primary myoblasts isolated from various strains (as indicated in figure legends) were plated in 96-well plates or chamber slides and treated as described above. The cells were fixed with ice cold 4%PFA in PBS for 10 minutes, permeabilized with 0.5% Triton X-100 in PBS for 6 minutes, and blocked in PBS with 0.025% Tween20, 10% normal horse serum and 10% normal goat serum for 1 hour at room temperature. Primary antibody incubation was done in blocking buffer overnight at 4 degrees, with the following antibodies: Myosin Heavy Chain (MyHC, MF20, DSHB hybridoma supernatant 1:10, or MY-32 ABCAM ab51263 1:400), Myogenin (MYOG sc-13137 SCBT 1:200), pHistone 3 (PH3, ab47297 ABCAM 1:1000), Ki-67 (Cell Marque #275R), RYR (ab2868 ABCAM 1:100), and pCaMKII (MERCK SAB4504356 1:100). Cells were washed 3 times in PBS with 0.025% Tween20 and then incubated with appropriate secondary antibodies in PBS for 1 hour. Where indicated, nuclei were either labeled with DAPI (MERCK D9542, 5ug/ml) or Hoechst 33342 (Thermo scientific #62249, 1:2000). Cells were imaged using the Nikon Eclipse Ti2 microscope (further described in microscopy section). All analysis was performed on at least 1000 nuclei. For fixed cells following the time-course with ERKi or DM (Figure 1B), images were captured with an inverted Olympus IX83 microscope (details in microscopy section). All imaging analysis were performed on at least 1000 cells.

Generation of retroviruses and transduction for live-cell imaging

The pBabe-puro and pBabe-GFPfarn plasmids were purchased from Addgene (Plasmid #1764 and #21836, respectively). The pBABE-CFP-NLS plasmid was constructed by replacing the PuroR gene from the pBABE-Puro with the coding DNA sequence of cyan fluorescent protein (CFP) fused to a tandem repeat of a nuclear localization signal (NLS) at the C-terminal (2 x PKKKRKV). Forward and Reverse DNA primers used for restriction free (RF) cloning of CFP-2xNLS from pCDNA3.1-CFPnls (Avinoam et al., 2011) into a pBABE vector are listed in Table S2. pBABE-dsRed was constructed in a similar manner (see Table S2 for primer information). 4hrs prior to transfection, 3×10^6 cells Platinum E Cells (Cell Biolabs) were seeded in 100-mm culture dish. 10μg of appropriate retroviral plasmid DNA (indicated in figure/video legends) was transfected using FuGENE 6 (Roche). Viral suspension was collected from the conditioned media 48hrs post transfection. The media was centrifuged (1000 RCF/10mins) to remove cell debris. The clarified viral suspension was used to transduce primary myoblasts. First passage primary myoblasts were seeded at 30,000 cells per well of a 6-well plate, 48 hrs prior to transduction using Polybrene (6μg/mL) (Merck: #TR 1003-G) as a transduction reagent. 1.5hrs after infection, viral suspension was removed, cells were washed with PBS, and fresh Bioamf-2 culture media was added to cells. 24hrs following transfection, cells were trypsinized and seeded in 8-chamber slide (Ibidi #80826) at a density of 20,000/well and allowed to attach. The following day, proliferation media was replaced with the appropriate treatment condition and imaging began (time of initiation and duration are shown in figure legends).

Spinning-disc confocal microscopy

Live cell imaging (37°C, with 5% CO2) was performed using Olympus IX83 fluorescence microscope controlled via VisiView software (Visitron Systems GmbH) and equipped with CoolLED pE-4000 light source (CoolLED Ltd., UK), an PLAPON60XOSC2 NA 1.4 oil immersion objective, and a Prime 95B sCMOS camera (Photometrics). Fluorescence excitation and emission were detected using filter-sets 488 nm and 525/50 nm for GFP, 561nm and 609/54 nm for mCherry.

Cell Discoverer 7-Zeiss microscopy

Fixed samples (Figure 1B) were imaged using Cell discoverer 7-Zaiss inverted in widefield mode with s CMOS 702 camera Carl Zeiss Ltd. Images were acquired using a ZEISS Plan-APOCHROMAT 20x / 0.95 Autocorr Objective. ZEN blue software 3.1 was used for image acquisition using AF647 for the acquisition of the MyHC signal and DAPI for the nuclei. If necessary, linear adjustments to brightness and contrast were applied using ImageJ v1.52 software (Schneider et al., 2012).

Nikon Eclipse Ti2 microscopy

Fixed samples (Figures 2 and 3) were imaged using the Nikon Eclipse Ti2 microscope and NIS-Elements imaging software ver.5.11.00. using a 10x objective for the acquisition of MyHC, MYOG, KI-67, pH3 and DAPI staining. If necessary, linear adjustment to brightness and contrast were applied using Photoshop. Live-imaging of tdTomato expressing myoblasts (Videos S1 and S2) were imaged using the Nikon Eclipse Ti2 microscope and NIS-elements software, using a 10x objective. linear adjustments to brightness and contrast were applied using ImageJ v1.52 software (Schneider et al., 2012).

Quantification of fusion index, MYOG nuclear localization, and migration rate

Following immunostaining and imaging, a fusion index was quantified by manually identifying nuclei found in a MyHC positive cell with at least 2 nuclei. Then the values were expressed as a percentage of the total nuclei per field. Briefly, in Figures where fusion index is stratified into subgroups of fiber size, the nuclei number in MyHC positive cell was manually quantified in a given field and stratified into groups of mononucleated, bi-nucleated myotubes, myotubes with 3-10 nuclei and myotubes with greater than 10 nuclei. For myotube growth curves, LifeAct-EGFP; nTnG reporter primary myoblasts underwent time-lapse imaging beginning at 8 hours after treatment and followed until 23 hours. Fields were analyzed hourly, and nuclei per cell was quantified and stratified into mononucleated, bi-nucleated, trinucleated and cells with ≥ 4 nuclei. In later experiments nuclei were segmented and count using the Cellpose software (Stringer et al., 2021) together with a home-made python script to match the nuclei to the cells. Nuclei positive after MYOG immunofluorescence staining were segmented and overlapped computationally over an image of the total segmented nuclei for each field, and the percent of MYOG positive out of the total was nuclei calculated. Cell migration rate was calculated by tracking the nuclei and calculating their displacement in x and y between time frames using a home-made script.

Data-driven cell fusion simulations

For each experiment we defined a matched “shadow” simulation that compared the experimental fusion dynamics to a scenario where cell-cell fusion occurred randomly. The input for the “shadow” simulation was the observed distribution of multinucleated cells in each time frame. This included the number of cells with a single, pair, triplet or quartette-or-more nuclei that were manually annotated with a time resolution of 60 minutes intervals between consecutive measurements. The estimated number of fusion events per time interval was calculated as the difference between the weighted accumulated number of multinucleated cells $\sum_{i=2}^{i=4}[(C_t(i) - C_{t-1}(i)) * (i-1)]$, where i is the number of nuclei in a multinucleated cell, t is the time interval and Ct(i) is the number of cells with i nuclei at time interval t. We assumed that the number of cells remain constant throughout the experiment. The input for the simulation included (1) N - the number of nuclei determined at the onset of the experiment, where each of the cells had exactly one nucleus. And (2) N_fusion - the list of estimated fusion events per time interval. For each time interval t, we simulated N_fusion(t) fusion events by randomly selecting two cells and fusing them, generating one cell with the joint number of nuclei for the next simulation round. For each time interval, we recorded the probability of a nucleus to be part of a 4-nuclei cells, i.e., what is the fraction of nuclei in a multinucleated cell that contains 4 or more nuclei. This fraction was used as a measure to compare experiments to simulations. Due to annotation limitations, we considered multinucleated cells that contained 4 nuclei. This means that a multinucleated cell with

more than 4 nuclei was annotated as a 4-nuclei cell. On the one hand, this limitation had implications in the calculations of the estimated number of fusions - which was a lower bound to the true number of fusion events. On the other hand, the calculated probability for a nucleus to take part in a 4-nucleated cell was also a lower bound to the true probability. This double lower bound effect is expected to cancel each other and also takes place only in the later stages of an experiment.

Statistical significance for each experiment was calculated using a Bootstrapping approach. For each experiment we performed 1000 simulations. For each time interval in each simulation, we recorded whether the probability of a nucleus to be in a 4-multinucleated cell was equal or exceeded the experimental observation. The p-value was defined as the probability for a simulation to exceed the experiment with this measure. We used a cutoff threshold $\leq$ 0.05 (50 simulations out of 1000 for each experiment) to reject the null hypothesis of random fusions. Importantly, this assessment provides a p-value for each time interval in each experiment. As a more realistic scenario we considered the possibility that the probability of selecting a cell for fusion was proportional to the number of nuclei within it. This followed the simplistic assumption that the area of a n-nucleated cell is n times the size of a single-nucleated cell. Thus, simulating the situation where a cell fuses randomly, but its chance of bumping-and-fusing into another cell is dependent on its area.

Quantitative real-time PCR (qRT-PCR)

Total RNA was isolated using Tri-Reagent (MERCK) according to the manufacturer's instructions. cDNA was synthesized with the High-Capacity cDNA Reverse Transcription Kit (Applied Biosystems) according to the manufacturer's instructions. qRT-PCR was performed with SYBR green PCR Master Mix (Applied Biosystems) using the StepOnePlus Real-time PCR system (Applied Biosystems). Values for specific genes were normalize to either *Gapdh* or *Hprt* housekeeping control as indicated in Figure legend. Expression was calculated using the ddCT method. Primer sequences are listed in Table S1.

Western Blot analysis

Cultured cells and whole tissues extracts were prepared with RIPA buffer supplemented with protease inhibitor cocktail (MERCK P8340), and phosphatase inhibitor cocktails (MERCK P5726 and P0044). Western blotting was performed using the Mini-PROTEAN Tetra Cell electrophoresis system, and transferred to PVDF membranes. The following primary antibodies concentrations were used p-CAMKII 1:1000 (Abcam ab182647), CaMKII 1:1000 (Cell Signaling 3362), GAPDH 1:10,000 (Abcam ab181602), p-ERK1/2 1:20,000 (MERCK M9692), ERK1/2 1:40,000 (MERCK M5670), p-RXR 1:1000 (Affinity Biosciences), RXR antibody 1:200 (SCBT sc-553), p-RYR 1:2000(Abcam ab59225), RYR 1:1000 (ab2868), p-Rac1 1:1000 (Millipore 07-896-I) Rac1 1:1000 (Millipore 05-389), (and Vinculin (provided by Benny Geiger, Weizmann Institute of Science). Horseradish peroxidase conjugated secondary anti-mouse, anti-rabbit or anti-goat was used to detect proteins (Jackson Immunology). Western blots were imaged using the Chemidoc Multiplex system (Bio-rad) and Image Lab software (Bio-rad).

Co-immunoprecipitation (Co-IP)

Primary myoblasts derived from gastrocnemius muscle were pooled from 10 mice and plated on 15cm dishes and allowed to adhere for 24 hours. The following day, Bio-Amf2 media was replaced supplemented either with DMSO or 1μm SCH772984. Cells were treated for 4 hours, and then nuclear lysates were prepared according to the instructions of the Universal Magnetic Co-IP KIT (Active Motif cat#54002). 1mg of protein was used to immunoprecipitate ERK1/2using 2ug of ERK1/2Antibody (MERCK M7927). Rabbit IgG was used as a control. Reactions were resuspended in 2x Sample buffer with DTT and loaded onto a 12% Tris-glycine SDS-page gel. 1% of original volume of lysate loaded into IP reaction was loaded into the gel as input control. Membranes were blotted with RXR antibody (SCBT sc-553).

Cloning and expression of CaMKII adenovirus for fusion assay

CaMKII-δ cDNA was PCR amplified from mouse primary myoblasts using primers, *CAMK2D-F* and *CAMK2D-R* (all cloning primer sequences are available in Table S2), designed against published CaMKII-δ sequences, and ligated into the PGEM-T-easy cloning system (Promega), and sequence validated. The T287V mutation was introduced by PCR assembly. A 909bp upstream PCR fragment was amplified with primer sequences designed to incorporate a XhoI site and FLAG tag at the N-terminus of CAMK2D and a the T287V mutation, using primers *XhoI-FLAG-CAMK2D-F* and *CAMK2D-T287V-IN-R*. The 640bp downstream PCR fragment was similarly amplified with a primer to introduce the T287V mutation and a BamHI site using the primers *CAMK2D-T287V-IN-F*: and *CAMK2D-BamHI-R*. Both PCR fragments were used as template for an assembly PCR reaction with *XhoI-FLAG-CAMK2D-F* and *CAMK2D-BamHI-R* primers to generate a 1525 bp product, which was ligated back into PGEM. Similarly, the WT CAMK2D was amplified with the same primers to incorporate the FLAG-tag and ligated back into PGEM. The 1525bp FLAG-CAMK2D^{WT} and FLAG-CAMK2D^{T287V} fragments were digested out of PGEM with BaMHI and XhoI and ligated into pEGFP-C1 (Clontech). A 2865 bp product EGFP-FLAG-CAMK2D^{WT} or EGFP-FLAG- CAMK2D^{T287V} was digested out using KPNI and ECORV and inserted into RedTrackCMV (addgene plasmid #50957). RedTrack-CMV-EGFP-FLAG-CAMK2D^{WT} (Ad-CaMK2D^{WT}), RedTrack-CMV-EGFP-FLAG-CAMK2D^{T287V}(Ad-CaMK2D^{T287V}), and empty RedTrack-CMV (Ad-Ctrl), vector were used as template to grow adenovirus using the Adeasy system as previously described (Luo et al., 2007). Myoblasts were infected with crude adenoviral lysate at an MOI of 100 at the time of plating (reverse infection) in BioAmf2 media. Following overnight incubation, the cells were washed once with warm DM and were incubated for 72 hours in DM and number of nuclei per fiber was quantified.

Myomaker plasmid construct and overexpression fusion assay

To generate pBabe-Mymk-CFPnls, the CDS sequence of murine MYMK (Millay et al., 2013) was subcloned in the MCS region of pBabe-CFPnls plasmid using restriction free cloning. Primer sequences are provided in in Table S2. Retroviruses were generated as described above. Myoblasts were seeded at 7x10^{3} per well of 96 well. The following morning cells were infected with viral prep supernatants together with polybrene (6ng/μL) for 1 hour, then replaced with fresh growth media, then after 8 hours the media was changed according to indicated conditions. Cells were fixed and stained at 18 hours post treatment.

CellPress
OPEN ACCESS

Histology and CSA quantification

14 days post CTX induced reinjury, muscles were excised and fixed in 4% PFA, embedded in paraffin, and sectioned. Muscles were cut transversely in the center and cut into serial sections at 0.3mm intervals. For analysis of muscle fiber cross-sectional area (CSA), sections were permeabilized and stained with WGA and DAPI. The entire muscle transverse section of WT and scDKO mice taken at identical locations within the muscle were imaged using the Nikon at 10x. CSA was quantified using the Open-CSAM, semi-automated analysis tool with ImageJ (Desgeorges et al., 2019). Each field was evaluated for accuracy and manually corrected. At least 9,000 fibers/mouse were measured.

Proximity ligation assay

Primary myoblasts were isolated from Wiltype or mTmG expressing mice as described above. Following treatment and fixation with 4% PFA, PLA was performed using the Duolink Proximity Ligation Assay (MERCK) according to manufacturer's instructions. Validation studies with individual antibodies were performed (not shown) to demonstrate specificity of the PLA signal. For the CaMKII:MYMK PLA, rabbit-anti-TMEM8C (MERCK HPA051846,1:50) and mouse-anti-CaMKII (SCBT sc-5306, 1:50) were used. For the Rac1:CaMKII PLA, rabbit anti-CaMKII (AB52476 1:100) and mouse-anti-Rac1(Millipore 05-389 1:100) were used. Where indicated, phalloidin-488 was used (ab176753) and DAPI (MERCK D9542, 5ug/ml

Statistical analysis

Sample size was chosen empirically following previous experience in the assessment of experimental variability. Generally, all experiments were carried out with $n \geq 3$ biological replicates. The analyzed animal numbers or cells per groups are described in the respective figure legends. All animals were matched by age and gender, and cells harvested from mice of similar age. Animals were genotyped before and after completion of the experiment and were caged together and treated in the same way. Statistical analysis was carried out using Prism software. Whenever comparing between two conditions, data was analyzed with two tailed student's t-test. If comparing more than two conditions, ANOVA analysis with multiple comparisons was executed. In all Figures, measurements are reported as mean of multiple biological repeats, and the error bars denote SEM, unless otherwise specified in the figure legend. Throughout the study, threshold for statistical significance was considered for p-values ≤ 0.05, denoted by one asterisk (*), two (**) if $P \leq 0.01$, three (***) if $P < 0.001$ and four (****) if $P \leq 0.001$.

Developmental Cell, Volume *56*

Supplemental information

ERK1/2 inhibition promotes robust myotube growth via CaMKII activation resulting in myoblast-to-myotube fusion

Tamar Eigler, Giulia Zarfati, Emmanuel Amzallag, Sansrity Sinha, Nadav Segev, Yishaia Zabary, Assaf Zaritsky, Avraham Shakked, Kfir-Baruch Umansky, Eyal D. Schejter, Douglas P. Millay, Eldad Tzahor, and Ori Avinoam

Supplemental Information

ERK1/2 inhibition promotes robust myotube growth via CaMKII activation resulting in myoblast-to-myotube fusion

Tamar Eigler[1], Giulia Zarfati[2], Emmanuel Amzallag[1], Sansrity Sinha[2], Nadav Segev[2], Yishaia Zabary[3], Assaf Zaritsky[3], Avraham Shakked[1], Kfir-Baruch Umansky[1], Eyal D. Schejter[4], Douglas P. Millay[5,6], Eldad Tzahor[1*†] and Ori Avinoam[2*]

Affiliations:

[1]Department of Molecular Cell Biology, Weizmann Institute of Science, Rehovot, Israel.

[2]Department of Biomolecular Sciences, Weizmann Institute of Science, Rehovot, Israel.

[3]Department of Software & Information Systems Engineering, Ben Gurion University, Be'er Sheva, Israel.

[4]Department of Molecular Genetics, Weizmann Institute of Science, Rehovot, Israel.

[5]Division of Molecular Cardiovascular Biology, Cincinnati Children's Hospital Medical Center, Cincinnati, Ohio, USA.

[6]Department of Pediatrics, University of Cincinnati College of Medicine, Cincinnati, Ohio, USA.

* Both authors share corresponding authorship: eldad.tzahor@weizmann.ac.il and ori.avinoam@weizmann.ac.il

† Lead contact

Supplemental Figure 1: ERKi inhibition in DM enhances differentiation and fusion, related to figure 1

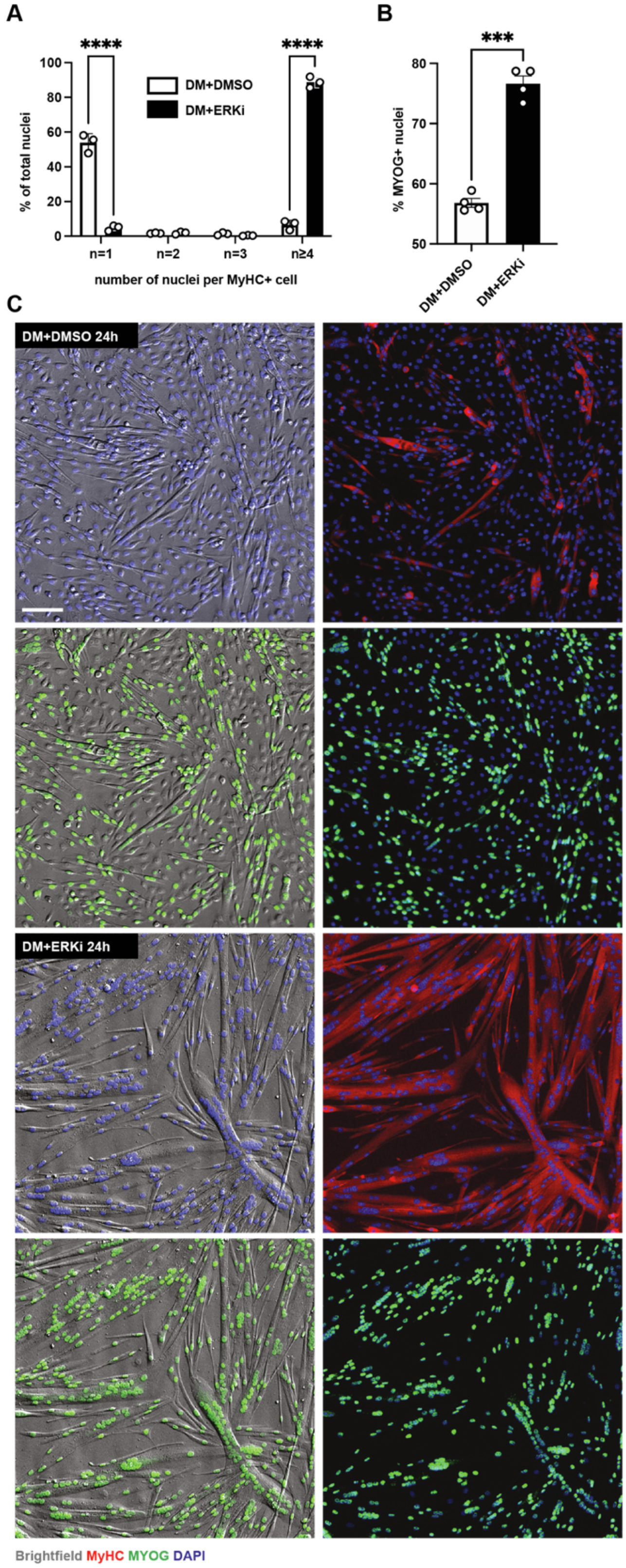

Supplemental Figure 1:

(A) Stratified fusion index of myoblasts co-treated with DM and ERKi , compared to DM treatment alone for 48 hours. (**B**) Quantification of MYOG positive nuclei per field as shown in **C**, using a semi-automated image analysis script to segment and overlap MyoG positive nuclei with total nuclei in an unbiased manner. See materials and methods for details. (**C**) Representative images of the IF staining for MYOG (green) and their correlation with nuclei (blue), and MyHC (red). Scale bars, 100 μm.

Supplemental Figure 2: ERKi induced fusion is not recapitulated by simulation studies of random fusion or weighted fusion, related to Figure 2

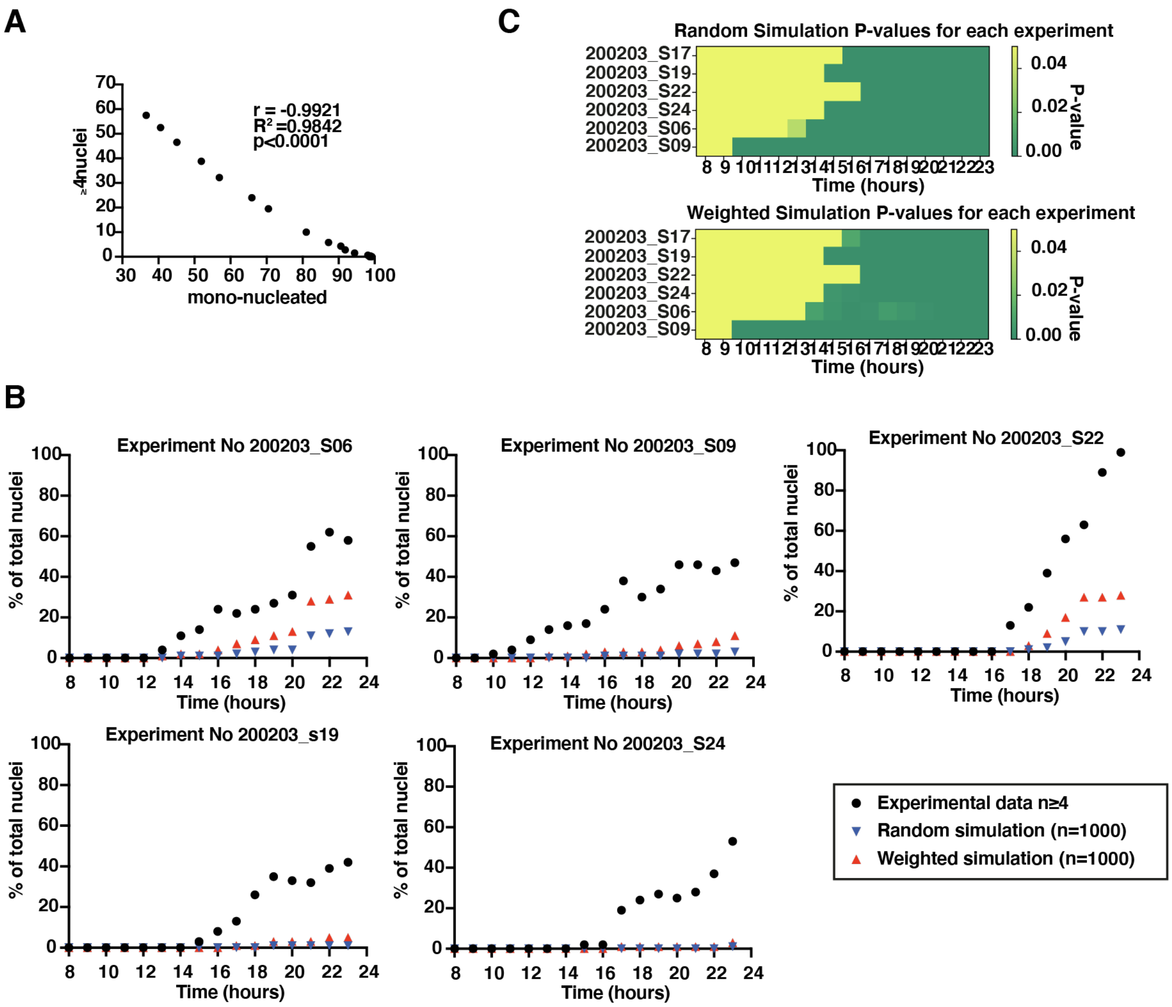

Supplemental Figure 2:

(**A**) Inverse correlation of mono-nucleated cells and multinucleated cells (≥4) from **Figure 2A**. (**B)** Data-driven simulations for five additional experiments establish that the fraction of nuclei present in multinucleated cells cannot be explained by random or weighted probabilities. See materials and methods and Figure 2C for details. (**C**) Corresponding p-values calculated with a bootstrapping approach for the fraction of nuclei in multinucleated cells for each time point in the experiment versus random (Top) and weighted simulations (Bottom). Significance level of 0.05 or lower was achieved after 10-17 hours from the onset of the experiment.

Supplemental Figure 3: Myoblast differentiation upon ERKi is not affected by blockade of calcium availability, related to Figure 3

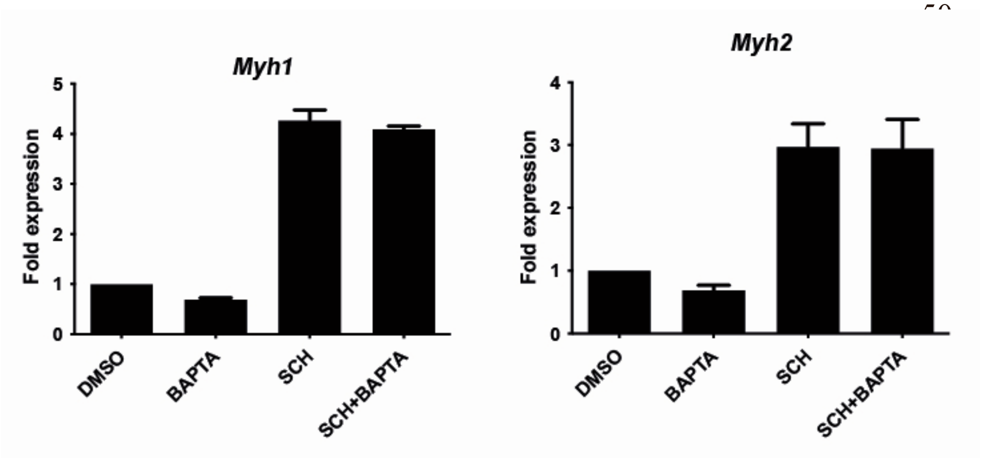

Supplemental Figure 3

Quantitative Realtime PCR analysis of the fold change in gene expression of myosin heavy chain 1 (Myh1) and 2 (Myh2) at 24 hours after treatment with either control (DMSO), 10uM BAPTA-AM, 1uM ERKi, or the combination of both ERKi and BAPTA-AM. Data is represents the mean +/- SEM of 3 biological repeats.

Supplemental figure 4. Inhibition of RXR and RYR inhibits fusion of DM treated myoblasts, related to Figure 3

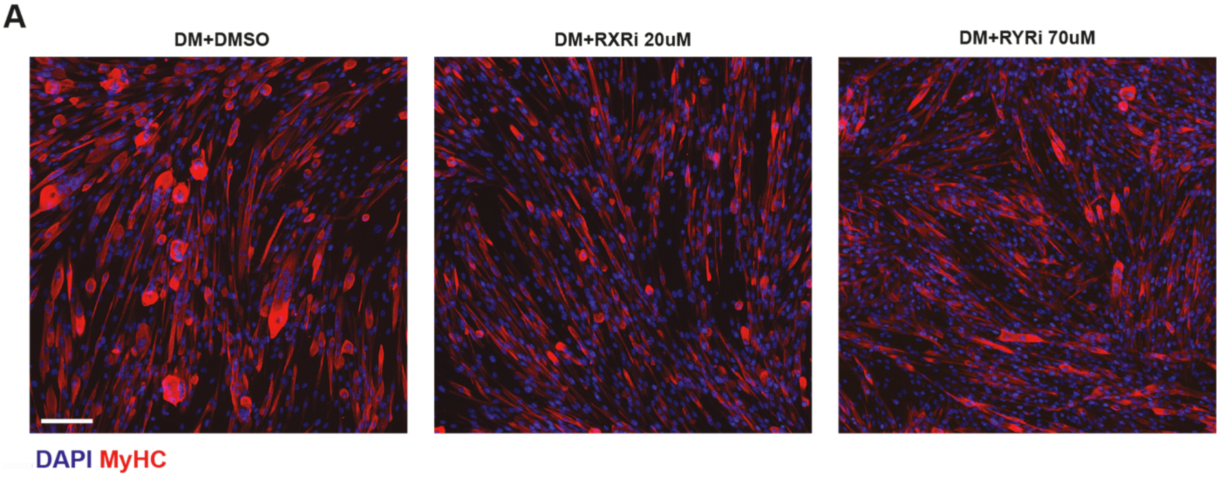

Supplemental figure 4:

(**A**) Representative IF staining of MyHC (red) and nuclei (DAPI, blue) for DMSO (ctrl), RXRi, or RYRi treated myoblasts grown in DM for 48 hours, showing their effect on fusion independent of ERKi. Scale bars, 100 μm.

Supplemental Figure 5: Evaluation of CaMKII activity downstream to ERKi, and its requirement for fusion, related to Figure 4

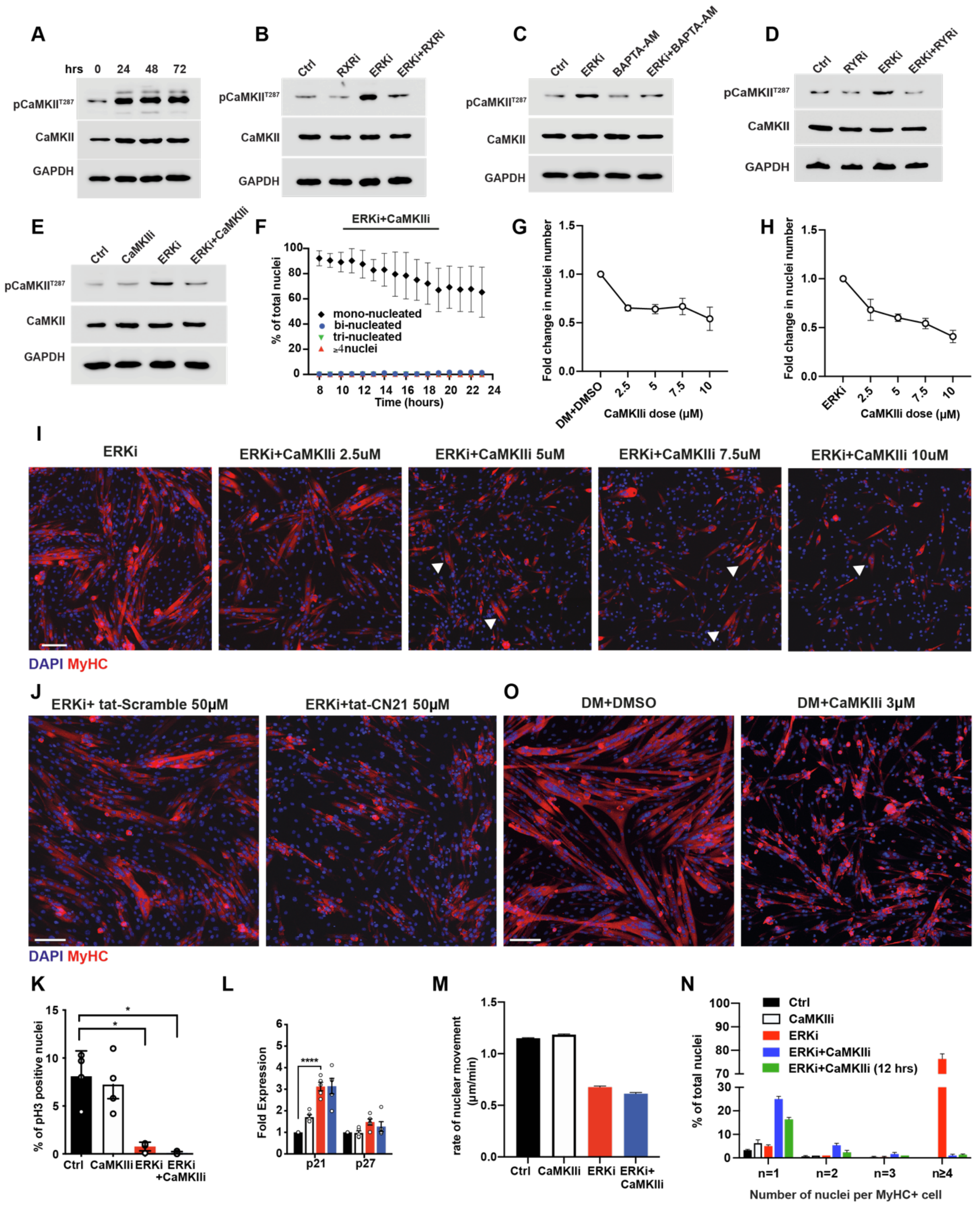

Supplemental Figure 5:

(**A**) Representative WB showing the activation of CaMKII in myoblasts treated with DM over 72 hours. (**B**) Representative WB showing the activation of CaMKII in myoblasts, treated with DMSO (Ctrl), 1 μM ERKi, 20 μM HX531(RXRi), or cotreated with ERKi and RXRi. (**C**) Representative WB showing the activation of CaMKII in myoblasts, treated with DMSO (Ctrl), 1 μM ERKi, 10μm BAPTA-AM, or cotreated with ERKi and BAPTA-AM. (**D**) Representative WBs showing CaMKII activation in myoblasts following 24 hours treatment with DMSO (Ctrl), 1 μM ERKi, Dantrolene 50 μM (RYRi), or cotreated with ERKi and RYRi. (**E**) Representative WB showing CaMKII activation of myoblasts treated with DMSO (Ctrl), 1 μM SCH772984 (ERKi), KN93 5 μM (CaMKIIi), or co-treated with ERKi and CaMKIIi at 24hrs post treatment. (**F**) Hourly fusion index following co-treatment of ERKi and CaMKIIi, showing the distribution of mono-, bi-, tri- and multi- nucleated (n≥4) cells. Total number of cells analyzed n=12,325. (**G**) Dose response of CaMKIIi treatment compared to treatment with DMSO, reported as change in nuclei number per field (**H**) Dose response of co-treatment with CaMKIIi compared to ERKi treatment alone, reported as change in nuclei number per field. (**I**) Representative IF images of MyHC (red) and nuclei (DAPI, blue) staining for the co-treatment dose response of CaMKIIi together with ERKi (1μm) at 24 hours post treatment. Arrows indicate bi or tri-nucleated cells that were still able to form. (**J**) Representative IF images of myoblasts co-treated with ERKi together with scrambled peptide (TAT-Scramble) or CaMKII peptide inhibitor (TAT-CN21) at 24 hours post treatment, MyHC (red) and nuclei (DAPI, blue). (**K**) Quantification of pH3 positivity following treatment with DMSO (Ctrl), 1 μM SCH772984 (ERKi), KN93 5 μM (CaMKIIi), or cotreated with ERKi and CaMKIIi at 24hrs post treatment (**L**) qRT-PCR expression analysis of *p21* and *p27* following co-treatment with ERKi and CaMKII, normalized to *Hprt*. Values are

expressed as fold change from that of DMSO (Ctrl). (**M**) Quantification of cell motility of myoblasts treated with DMSO (Ctrl), 1 μM SCH772984 (ERKi), KN93 5 μM (CaMKIIi), or cotreated with ERKi and CaMKIIi over a 24-hour period. (**N**) Stratified fusion index of cells which received a delayed co-treatment of CaMKII 12 hour following initial treatment with ERKi. (**O**) Representative IF images of myoblasts grown in DM or co-treated with CaMKIIi for 48 hours. All scale bars, 100 μm.

Supplemental Figure 6: RYR is exclusively localized to post-fusion myotubes and not to mononucleated cells, related to Figure 4

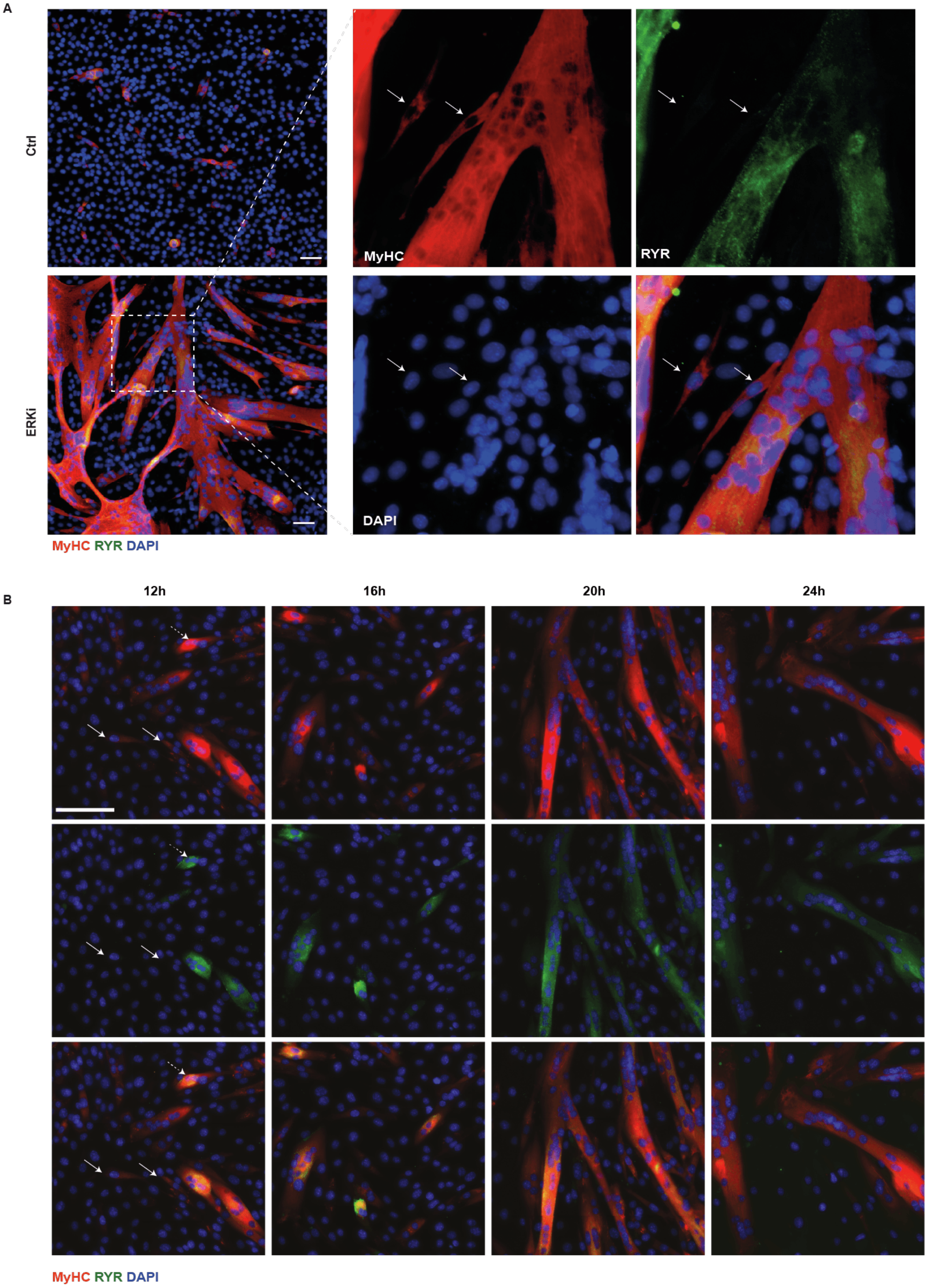

Supplemental Figure 6:

(**A**) Representative images of Ryanodine receptor (RYR) IF showing its localization in myotubes in Ctrl and ERKi treated cultures at 24hrs post-treatment. Indicated region in ERKi image is enlarged on right, showing the individual fluorescence channels and an overlay. Arrows indicate differentiated ($MyHC^{+}$) myoblasts lacking ryanodine receptor. (**B**) Representative IF images of RYR and MyHC at different time points after ERKi treatment showing that RYR is expressed in $MyHC^{+}$ multinucleated cells and mono nucleated cells (solid arrows). Dashed arrow indicates a bi-nucleated cell which is RYR positive. RYR (green), MyHC (red) and nuclei (DAPI, blue). Scale bars, 100 μm.

Supplemental Figure 7: Evaluation of successful PLA reactions, related to Figure 5

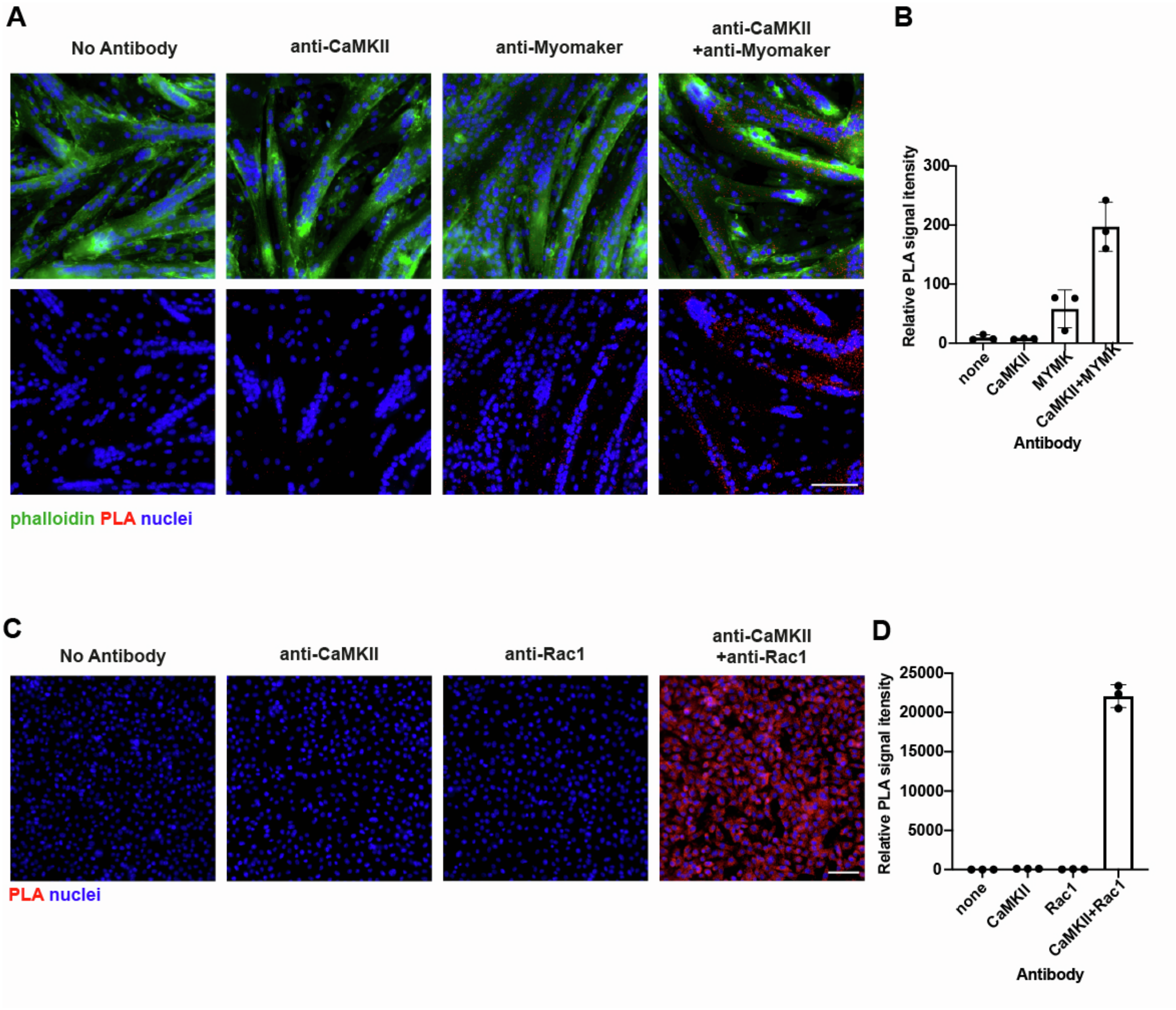

Supplemental Figure 7:

A) Negative controls for PLA interaction of Myomaker and CaMKII in ERKi induced myotubes. Top panel: Overlay of PLA signal (red), phalloidin (green) and DAPI (blue). Bottom panel: Overlay of the PLA signal (red) and DAPI (blue). Reactions were carried out either with no antibody or with the individual antibodies against Myomaker and CaMKII (anti-MYMK or anti-CaMKII respectively), or in comparison to the combination of both antibodies demonstrating the specificity of the PLA signal only when both antibodies are applied together. **B**) Quantification of the relative signal intensity of the PLA signal for each condition in **A**. The data represents the mean +/- SEM of 3 biological repeats. **C**) Negative controls for PLA interaction of Rac1 and CaMKII in proliferating myoblasts. Shown are the overlay of the PLA signal (red) and DAPI (blue). PLA reactions were carried out either with no antibody or with the individual antibodies against Rac1 or CaMKII (anti-Rac1 or anti-CaMKII respectively), or in comparison to the combination of both antibodies demonstrating the specificity of the PLA signal only when both antibodies are applied together. **D**) Quantification of the relative signal intensity of the PLA signal for each condition in **C**. The data represents the mean +/- SEM of 3 biological repeats. Scale bars = 100 μm.

Table 1: Primer list for qRT-PCR, related to Figure 1, 3, and 4

Gene	Forward Primer 5'→ 3'	Reverse Primer 5'→ 3'
Ryr1	ACGGAGAGAAAGTCATGGCG	ACTGATGGATTCCTGCAGCC
Ryr3	ACCAGCAGGAGCAAGTACG	GGGGTCGTGTCAAGTAGTCA
Orai1	GATCGGCCAGAGTTACTCCG	TGGGTAGTCATGGTCTGTGTC
Orai2	GACAGTCAGGCCTGGTCCC	CGGACCCAGTCTCGGTAATC
Stim1	CTTGCCCTGTGGCTTCTTTG	ATTCGGCAAAACTCTGCTTCG
Stim2	CTTGCGAGAACGGCTTTTTCG	GTACAGAGAGGAGGTGAGACTG
Itpr1	GGGTCCTGCTCCACTTGAC	CCACATCTTGGCTAGTAACCAG
Itpr2	TTCAGTTCCTATCGAGAGGATGT	GCTGATTGACGCAAGGTCG
Itpr3	GGGCGCAGAACAACGAGAT	GAAGTTTTGCAGGTCACGGTT
Atp2a1	TGTTTGTCCTATTTCGGGGTG	AATCCGCACAAGCAGGTCTTC
Atp2a2	TGGAACAACCCGGTAAGAGT	CACCAGGGGCATAATGAGCAG
Atp2a3	CGTCGCTTCTCGGTGACAG	AAGAGGTCCTCAAACTGCTCC
Pax7	CGGGTTCTGATTCCACATCT	CGACGAGGAAGGAGACAAGA
Myf5	ACGGCATGCCTGAATGTAAC	AGCTGGACACGGAGCTTTTA
Myog	GAAGCGCAGGCTCAAGAAAG	GCCGCGAGCAAATGATCTCC
MyoD	AACTGCTCTGATGGCATGATG	TGGAGATGCGCTCCACTATG
p27	CAGACGTAAACAGCTCCGAATTA	TCAGTGCTTATACAGGATGTCCA
p21	AGAGACAACGGCACACTTTG	CGGTGTCAGAGTCTAGGGGA
Mymk	GGGCTGTTCCATAGATGCTG	GGAGGCCATGGTCTACCTCT
Mymx	GTTAGAACTGGTGAGCAGGAG	CCATCGGGAGCAATGGAA
Gapdh	GGGTCCCAGCTTAGGTTCAT	CCAATACGGCCAAATCCGTT
Hprt	AGCGTCGTGATTAGCGATGA	GCAAGTCTTTCAGTCCTGTCC

Table 2: Primer list for cloning, related to Figure 2, 4, and 5

Cloning primer name:	Primer sequence 5'-->3'
CAMK2D-F	ATGGCTTCGACCACCACCT
CAMK2D-R	TTAGTTGATGGGTACTGTGG
XhoI-FLAG-CAMK2D-F	AGATCTCGAGCTCAAGATTACAAGGATGACGACGATAAGATGGCTTCGACCACCACCTGC
CAMK2D-T287V-IN-R:	AAGCAGTCTACATCCTCCTGCCTG
CAMK2D-BamHI-R	GGTGGATCCTCAGATGTTTTGCCACAAAGAGGT
CFP-F	GGCTTTTTTGGAGGCCTAGGCTTTTGCAAAAAGCTTACCATGGTGAGCAAGGGCGAG
CFP-R	CGGGTCGTGGGGCGGGCGTTATACCTTTCTCTTCTTTTTGGATCTACCTT
dsRED-F	TGAGGAGGCTTTTTTGGAGGCCTAGGCTTTTGCAAAAAGCTTACCATGGCCTCCTCCGAGAACG
dsRED-R	ACTGACACACATTCCACAGGGTCGACCTCAGACACAAGTGCAGCA
MYMK-F	GCCCTCACTCCTTCTCTAGGCGCCGATGGGGACAGTTGTAGCCA
MYMK-R	ACTGACACACATTCCACAGGGTCGACCTCAGACACAAGTGCAGCA
